# Transcriptomic benchmarking positions iPSC-Derived intestinal organoids as immature, non-malignant epithelial references for colorectal cancer tissue

**DOI:** 10.1016/j.bbrep.2026.102859

**Published:** 2026-11-03

**Authors:** Zeynep Büşra Aksoy, Recep Uyar, Hilal Özdağ Sevgili, Kamil Can Akçalı

**Affiliations:** a Ankara University Stem Cell Institute, Ankara, Türkiye; b Ankara University Institute of Health Sciences, Ankara, Türkiye; c Ankara University, Genetic Department, Institute of Forensic Medicine, Ankara, Türkiye; d Ankara University Biotechnology Institute, Ankara, Türkiye; e Ankara University School of Medicine, Biophysics Department, Ankara, Türkiye

**Keywords:** Colorectal cancer, Intestinal organoids, iPSC-derived organoids, RNA-seq, Transcriptomics, Epithelial differentiation, Tumor microenvironment

## Abstract

Primary colorectal cancer (CRC) biopsies and intestinal organoids are frequently compared to define tumor-associated epithelial alterations; however, these systems differ in malignant status, tissue composition, developmental state, and epithelial maturation. In this study, transcriptomic profiling of CRC biopsies, matched normal intestinal tissue, and iPSC-derived intestinal organoids (iIOs) was performed to define the biological axes separating primary tissue from organoid epithelial states. Transcriptome-wide analysis revealed strong separation between CRC biopsies and iIOs, with iIOs diverging from primary tissue more extensively than tumors differed from normal mucosa. Pathway enrichment and cell deconvolution showed that biopsies were dominated by immune, inflammatory, and stromal programs reflecting tissue complexity, whereas iIOs were enriched for proliferative, biosynthetic, and metabolic programs. Projection onto a malignancy axis placed iIOs at a neutral position, indicating that their divergence stems from developmental state rather than malignant transformation. Compared to normal mucosa, organoids reproducibly lost adult tissue-associated immune/stromal programs while gaining proliferative, fetal-like epithelial programs. Gut Cell Atlas scoring, crypt–surface positioning, and lineage deconvolution showed that iIOs retain intestinal epithelial identity but occupy a developmentally immature, crypt/progenitor-biased state dominated by transit-amplifying programs with limited differentiated lineage diversity. Metabolic module analysis and anabolism-proliferation correlation showed that elevated metabolic activity in iIOs, including biosynthetic and nucleotide synthesis programs. These reflect proliferation-coupled anabolism rather than malignancy-like metabolic reprogramming; the glycolysis-OXPHOS balance did not differ significantly between groups. Consequently, iIOs represent metabolically active, fetal-like, crypt/progenitor epithelial reference models rather than direct transcriptomic surrogates of adult intestinal mucosa or primary CRC tissue. These results clarify the utility and limitations of iIOs as epithelial reference models for colorectal cancer research.

## Highlights

• IIOs diverge from CRC biopsies more than tumors differ from normal mucosa.
• Biopsies are immune/stromal-dominant; iIOs are proliferative and biosynthetic.
• IIOs show a neutral malignancy-axis score, consistent with developmental origin.
• Elevated iIO metabolism reflects proliferation-coupled anabolism, not Warburg effect.

## Graphical abstract

**Figure:**
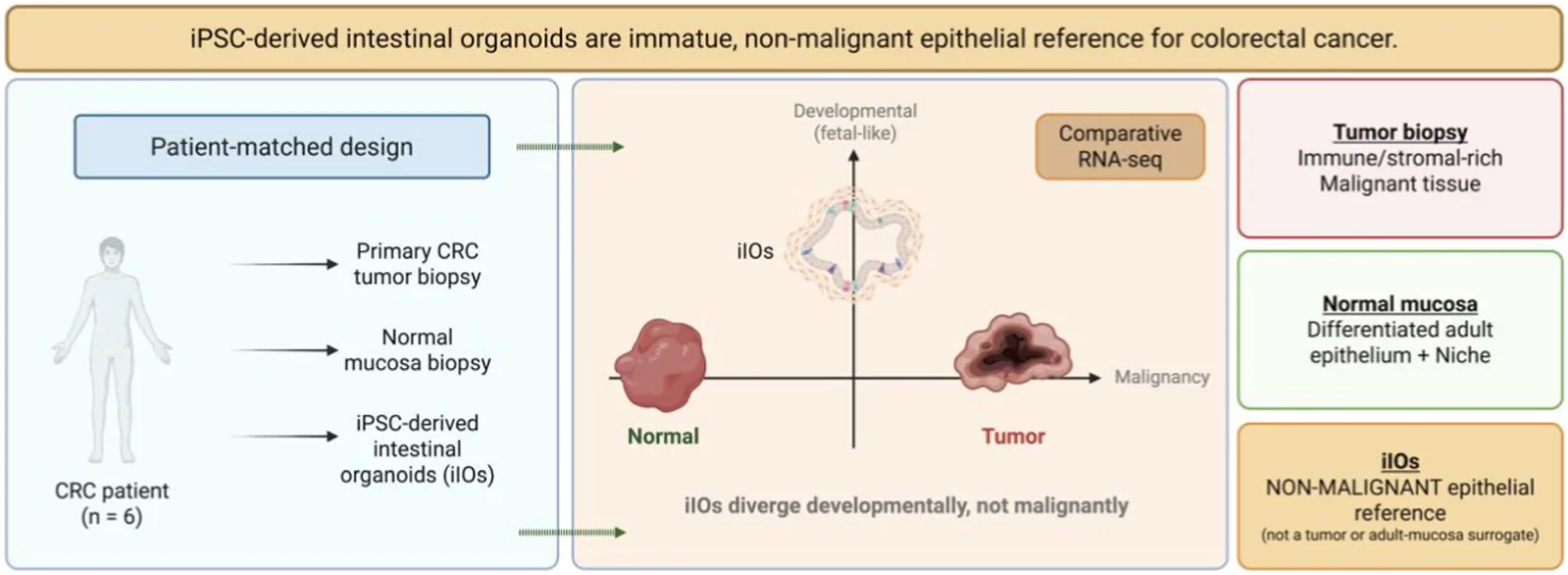

Created in BioRender. Doğan, E. (2026) https://BioRender.com/8c44clx; publication license was uploaded as supplementary file.

## 1 Introduction

Colorectal cancer (CRC) is one of the major malignancies with high incidence and mortality worldwide and continues to represent a significant public health burden. In CRC development, multilayered biological transformation processes are dynamically active, including not only uncontrolled cellular proliferation and disruption of tissue architecture, but also loss of cellular differentiation programs, metabolic reprogramming, epigenetic imbalance, and remodeling of the tumor microenvironment [1], [2], [3]. Therefore, in current approaches aimed at understanding CRC biology, elucidating the fundamental axes along which malignant tissue deviates from healthy intestinal epithelial organization, in addition to defining tumor-specific molecular alterations, may enable a better understanding of the more complex process of CRC development. Beyond tissue level alterations, CRC progression is also driven by complex molecular regulatory cascades, including competing endogenous RNA (ceRNA) networks involving long non-coding RNAs and microRNAs [4]. While such conventional *in vitro* (2D) and in vivo models have elucidated critical molecular axes and non-coding RNA regulatory networks in CRC, physiologically relevant 3D platforms are increasingly needed to capture the epithelial organization and developmental context of the intestine.

In healthy physiology, the intestinal epithelial tissue has an architecture in which polarity and barrier integrity are regulated along the crypt–villus axis, and which contains highly organized and functionally distinct cell populations [5], [6], [7]. The intestinal epithelial structure comprises a dynamic homeostatic system in which stem cells, transit-amplifying cells, and differentiated enterocytes, goblet cells, Paneth cells, and enteroendocrine cells are tightly coordinated [8], [9], [10]. However, in colorectal tumors, this hierarchical organization is markedly disrupted; tissue polarity is lost or substantially weakened, barrier function is impaired, cellular reserve balance is altered, and proliferative/oncogenic programs become dominant [11], [12], [13]. In addition, biopsies obtained from primary tumor tissue have shown that, because primary tumor biopsies contain not only the neoplastic epithelium but also stromal components such as fibroblasts, immune cells, vascular elements, and extracellular matrix, the transcriptomic profiles derived from these samples reflect not only intrinsic tumor cell signals but also a strong microenvironmental contribution [1], [14], [15], [16]. While this creates biological complexity in the interpretation of primary tumor data, it also makes the multicomponent nature of malignant tissue organization visible.

Developing *in vitro* organoid systems are increasingly being incorporated into the modeling of human tissue biology and disease mechanisms. They are now widely used in studies of human tissue biology and disease [17]. In particular, intestinal organoids (iIOs) derived from induced pluripotent stem cells (iPSCs) enable the reconstruction of key structural and functional features of the intestinal epithelium *in vitro* [18], [19]. Importantly, because iIOs are generated from reprogrammed somatic cells, they are non-malignant in origin and therefore distinct from patient-derived tumor organoids, which are established directly from tumor tissue and retain its malignant and genomic features [18], [19], [20]. In these models, epithelial polarity, multilineage differentiation, barrier formation, and tissue-specific gene expression are maintained *in vitro* [21]. Therefore, iIOs are highly valuable not only for investigating intestinal development and homeostasis, but also for systematically defining the ways in which the malignant tissue state diverges from healthy-like epithelial organization [22], [23]. Thus, although iIOs provide a valuable epithelial reference system, their relationship to primary CRC biopsies and matched healthy intestinal tissue must be interpreted within the context of tissue complexity, epithelial maturation, and developmental state.

In previous studies, the relationship between primary colorectal tumor biopsies and intestinal organoids has generally been reduced to a limited number of cancer-associated markers or specific functional readouts [18], [24], [25]. However, the distinction between primary tissue and iIOs extends beyond individual genes or isolated pathways. This requires looking beyond individual genes, at the transcriptome, the tissue's immune/stromal content, epithelial organization and lineage composition, developmental identity, and metabolic state together [26], [27], [28]. Such an approach is necessary to determine whether organoids primarily represent a healthy-like epithelial counterpart to tumor tissue, a developmental epithelial state, or a distinct culture-associated reference that captures only selected aspects of intestinal epithelial identity.

In this study, we generated and characterized iIOs and performed comparative transcriptomic profiling of primary CRC biopsies, matched normal intestinal tissue, and iIOs. We used differential expression analysis, gene set enrichment analysis, curated signature scoring, leading-edge gene analysis, cell population deconvolution, Gut Cell Atlas-based epithelial benchmarking, and metabolic module analysis to define the biological axes separating primary tissue from organoid states. Rather than assuming that iIOs are direct transcriptomic surrogates of adult healthy mucosa, we tested whether their divergence from CRC biopsies reflects malignancy-associated programs, primary tissue microenvironmental complexity, or developmental and culture-associated epithelial maturation state.

We hypothesized that primary CRC biopsies and iIOs occupy distinct transcriptomic states: CRC biopsies are expected to retain immune, stromal, and tissue-level microenvironmental programs, whereas iIOs are expected to represent a developmentally immature and crypt/progenitor-biased epithelial reference state. We additionally tested whether the metabolic state of iIOs reflects malignancy-associated reprogramming or proliferation-coupled anabolism, using curated metabolic module scoring and Warburg-index analysis. This study describes these differences across several biological layers to show how iIOs can, and cannot, be used as epithelial reference models for primary CRC tissue.

## 2 Materials and methods

### 2.1 Human participants and clinical samples

In this study, primary tumor biopsies from six colorectal cancer patients and iIOs samples generated from CRC patients (CRC-iIOs) were used. For additional comparative analyses, RNA-seq data from matched healthy biopsy samples obtained from non-tumor regions of the same patients were also evaluated. All procedures involving human samples were conducted in accordance with the Declaration of Helsinki and approved by the Lokman Hekim University Human Research Ethics Committee (Approval Date: 23/12/2021; Decision No: 2021/157). Informed consent was obtained from all participants or their legal guardians. The cohort comprised six patients with histologically confirmed colorectal adenocarcinoma (Table S4). Each patient contributed one primary tumor biopsy, one matched normal mucosal biopsy from non-tumor surgical margin of the same resection specimen, and one iIO generated from single iPSC clone derived from the patient's peripheral blood. All samples per patient were collected during the same surgical procedure, there are no technical replicates. All six iIO samples were generated under the same differentiation protocol and analyzed at day 120 (passage 20) in a single sequencing batch. Full patient and sample information are provided in Table S4.

### 2.2 Method details

#### 2.2.1 Generation of iPSCs from erythroid progenitor cells

Human induced pluripotent stem cells (iPSCs) were generated in-house and were not obtained commercially. iPSCs used in this study were generated from peripheral blood-derived erythroid progenitor cells using an integration-free Sendai virus reprogramming strategy adapted from our previously published protocol [29]. Briefly, peripheral blood mononuclear cells (PBMCs) were isolated from EDTA-anticoagulated blood samples by Ficoll density gradient centrifugation. Mononuclear cells were washed with DPBS and IMDM, counted, and plated for erythroid progenitor expansion. For erythroid enrichment, cells were cultured in StemSpan SFEM II supplemented with StemSpan Erythroid Expansion Supplement (erythroid expansion medium, EEM) for 7 days at 37°C and 5% CO2, with media renewal on days 2, 4, and 6. Erythroid progenitor identity was confirmed by flow cytometry using CD71 and GlyA (Fig. S4 A-B). For reprogramming, erythroid progenitor cells were transduced with CytoTune 2.0 Sendai viral vectors encoding OCT4, SOX2, KLF4, and c-MYC. Cells were resuspended at 1 × 10^5 cells/mL in EEM and transduced using a 5:5:3 MOI of hKOS:hKlf4:hcMyc. To enhance reprogramming efficiency, transduced cells were centrifuged at 1000 × g for 30 min and then plated in 48-well plates. On the following day, cells were transferred onto vitronectin-coated 6-well plates. Reprogramming media were transitioned sequentially from EEM to TeSR-E7/ReproTeSR and then to Essential 8 medium. Early colonies appeared between days 7 and 10, and well-defined iPSC colonies were typically observed by day 15. Colonies were maintained on vitronectin in Essential 8 medium and passaged at least four times before clonal selection and downstream characterization. Pluripotency was validated by qRT-PCR analysis of pluripotency-associated genes, alkaline phosphatase live staining, and immunofluorescence staining for OCT4, SOX2, SSEA4, and TRA-1-60 using the PSC 4-marker immunocytochemistry panel (Fig. S4 C-D). In addition, trilineage differentiation potential was assessed through embryoid body formation followed by spontaneous differentiation and immunostaining for GATA4, Nestin, and Desmin as markers of endoderm, ectoderm, and mesoderm, respectively (Fig. S4 E–G). All six iPSC lines were confirmed mycoplasma-negative by PCR-based detection assay (Mycoplasma detection kit, MedChem Express, HY-K0552) prior to differentiation. The residual Sendai vector was confirmed by RT-PCR at passage 12 for all lines using the Cytotune 2.0 primer sets targeting four viral components: SeV genome (forward: 5′-GGA TCA CTA GGT GAT ATC GAG C-3′; reverse: 5′-ACC AGA CAA GAG TTT AAG AGA TAT GTA TC-3′), KOS (forward: 5′-ATG CAC CGC TAC GAC GTG AGC GC-3′; reverse: 5′-ACC TTG ACA ATC CTG ATG TGG-3′), KLF4 (forward: 5′-TTC CTG CAT GCC AGA GGA GCC C-3′; reverse: 5′-AAT GTA TCG AAG GTG CTC AA-3′), and c-MYC (forward: 5′-TAA CTG ACT AGC AGG CTT GTC G-3′; reverse: 5′-TCC ACA TAC AGT CCT GGA TGA TGA TG-3′). Characterization data in Fig. S4 are shown for one representative iPSC line; quality-control data for all six lines are provided in the Supplementary QC File All human samples were collected under approval from the Lokman Hekim University Human Research Ethics Committee (No. 2021/157) with informed consent.

#### 2.2.2 Differentiation of iPSCs into intestinal organoids

In this study, human iPSC-derived colon organoids were generated using our previously published three-step directed differentiation protocol [30]. Briefly, iPSCs were maintained on Vitronectin-coated 6-well plates in Essential 8 Medium at 37°C with 5% CO2. Upon reaching 80–90% confluency, definitive endoderm (DE) differentiation was initiated (Day 0) by replacing the culture medium with DE Medium I (RPMI 1640 supplemented with 3 μM CHIR99021, 100 ng/mL Activin A, and 2 mM GlutaMAX). On Day 1, CHIR99021 was removed, and cells were cultured in DE Medium II (RPMI 1640 with 100 ng/mL Activin A and 2 mM GlutaMAX) through Day 2. DE induction efficiency was confirmed on Day 2 by flow cytometric analysis, ensuring >70% positivity for CXCR4 and CD117. Hindgut endoderm differentiation was performed from Day 3 to Day 6 using Hindgut Endoderm Medium (RPMI 1640 supplemented with 3 μM CHIR99021, 500 ng/mL FGF4, 2% B27, and 2 mM GlutaMAX) with daily medium changes. Starting from Day 7, the culture was switched to Colonic Maintenance Medium (Advanced DMEM/F12 supplemented with 3 μM CHIR99021, 300 nM LDN193189, 100 ng/mL EGF, 2% B27, and 2 mM GlutaMAX). Spontaneously budding hindgut spheroids were collected without centrifugation between Days 8 and 20, using Anti-Adherence Solution-prewetted tips. Collected spheroids were allowed to settle by gravity, embedded in Growth Factor Reduced Matrigel domes (40–100 μL), and polymerized at 37°C. Domes were overlaid with Colonic Maintenance Medium supplemented with 10 μM Y-27632 for the initial 48 h. Cultures were fed twice a week and passaged weekly by gentle mechanical dissociation following cold DMEM/F12-mediated Matrigel depolymerization.

#### 2.2.3 Flow cytometry

Definitive endoderm differentiation efficiency was evaluated by flow cytometry using CXCR4 and CD117 surface markers. Cells were harvested on day 3 of differentiation and centrifuged at 300 × g for 10 min. All subsequent staining steps were performed on ice or at 4°C. The cell pellet was resuspended in ice-cold staining buffer consisting of 1× PBS supplemented with 5% BSA and 0.05% sodium azide. An unstained control and the corresponding isotype controls were included for gate setting and background correction.

For each staining reaction, approximately 5 × 10^4^ cells were resuspended in 100 μL staining buffer and incubated with fluorochrome-conjugated antibodies for 30 min at 4°C in the dark. Cells were stained with fluorochrome-conjugated antibodies against PDGFRa, CXCR4 and CD117 or with the corresponding FITC mouse IgM and PE mouse IgG2a κ isotype controls, according to the experimental panel to confirm definitive endoderm differentiation. Following incubation, cells were washed with 1 mL of 1× PBS and centrifuged at 300 × g for 10 min. The final cell pellet was resuspended in 200 μL of 1× PBS before acquisition.

Flow cytometric acquisition was performed using an ACEA NovoCyte flow cytometer. Data were analyzed by first excluding debris based on forward and side scatter properties, followed by singlet gating. Fluorescence gates were established using unstained and isotype control samples. The percentage of marker-positive cells was calculated relative to the gated viable cell population. The fluorophore-conjugated antibodies used for flow cytometry are listed in the Supplementary Table S2.

#### 2.2.4 Flow cytometry gating strategy

Flow cytometry data were analyzed using a sequential gating strategy. First, debris and very small particles were excluded based on forward scatter area and side scatter area properties. The main cell population was selected on the FSC-A versus SSC-A plot and defined as the P5 gate. To exclude doublets and cell aggregates, cells within the P5 gate were further analyzed using FSC-A versus FSC-H, and the singlet population was selected as the P7 gate. Definitive endoderm marker expression was then evaluated within the P5/P7 gated singlet population using a two-parameter quadrant plot of CD117-FITC versus CXCR4-PE. Quadrant thresholds were determined using unstained and/or control iPSC samples. Cells positive for both CD117 and CXCR4 were considered definitive endoderm-like cells. The percentage of CD117^+^/CXCR4^+^ cells was calculated relative to the gated singlet population. Flow cytometry analysis demonstrated an increase in the CD117^+^/CXCR4^+^ double-positive population following definitive endoderm differentiation.

#### 2.2.5 Immunofluorescence staining

Immunofluorescence staining was performed to evaluate pluripotency, intestinal lineage marker expression, and spontaneous differentiation capacity. Cells were cultured on vitronectin-coated glass-bottom dishes or chamber slides until they reached the appropriate morphology. Cells were washed with 1× PBS and fixed with 4% paraformaldehyde for 15–20 min at room temperature. After fixation, samples were washed three times with PBS.

For intracellular staining, cells were permeabilized with 0.1% Triton X-100 in PBS for 10–15 min at room temperature. Non-specific binding was blocked using blocking buffer containing BSA or serum in PBS for 1 h at room temperature. Cells were then incubated with primary antibodies diluted in blocking buffer overnight at 4°C. Pluripotency was assessed using antibodies against OCT4, SOX2, SSEA4, and TRA-1-60. To assess intestinal lineage and epithelial differentiation, iIOs were embedded into OCT and sectioned for 5 μm thickness. iIO sections were evaluated using antibodies against FOXA2, KLF5, Chromogranin A, SOX9, Lysozyme, Villin, and LGR5, depending on the experimental stage. Negative controls without primary antibody were included to assess non-specific secondary antibody binding and background fluorescence.

After primary antibody incubation, cells were washed three times with PBS and incubated with the appropriate fluorophore-conjugated secondary antibodies for 1 h at room temperature in the dark. Donkey anti-rabbit Alexa Fluor 555, goat anti-mouse IgG3 Alexa Fluor 488, donkey anti-rat Alexa Fluor 488, goat anti-mouse IgM Alexa Fluor 555, and goat anti-rabbit IgG Alexa Fluor 488 secondary antibodies were used according to the host species of the primary antibodies. Following secondary antibody incubation, cells were washed three times with PBS. Nuclei were counterstained with DAPI. Images were acquired using a confocal fluorescence microscope (LSM 880, Zeiss) under identical imaging settings within each staining set. The primary and secondary antibodies used for immunofluorescence staining are listed in the Supplementary Table S2.

#### 2.2.6 RT-qPCR

The total RNA was extracted by PureZol RNA Isolation Reagent (Bio-rad, 7326890) followed by isopropanol precipitation according to the standard protocols. Reverse transcription was performed with iScript cDNA Synthesis Kit (Bio-rad, 1708890). Quantitative real-time PCR was carried out using SsoAdvanced Universal SYBR Green Supermix (Bio-rad, 1725274). Gene expression was normalized to *β-Actin*. Primer sequences used for gene expression analysis are as following: *hBeta-Actin_forward* 5′-AAAATCTGGCACCACACCTTC-3′, *hBeta-Actin_*reverse 5′-AGCACAGCCTGGATAGCAAC-3’; *hEndo-OCT4_forward* 5′-AGTTTGTGCCAGGGTTTTTG-3′, *hEndo-OCT4_reverse* 5′-ACTTCACCTTCCCTCCAACC-3’; *hEndo-SOX2_forward* 5′-GGGAAATGGGAGGGGTGCAAAAGAGG-3′, *hEndo-SOX2_reverse* 5′-TTGCGTGAGTGTGGATGGGATTGGTG-3’; *hNANOG_forward* 5′-CTCTCCAACATCCTGAACCTC-3′, *hNANOG_reverse* 5′-ACACCATTGCTATTCTTCGG-3′, *hc-MYC_forward* 5′-GGATTCTCTGCTCTCCTCGAC-3′, *hc-MYC_reverse* 5′-CTTCCTCATCTTCTTGTTCCCT-3′, *hKLF4_forward* 5′-AAACCTACACAAAGAGTTCCCAT-3′, *hKLF4_reverse* 5′-TTCCATCCACAGCCGTCCCA-3′, *aSMA_forward* 5′-TGAAGATCAAGATCATTGCCC-3′, *aSMA_reverse* 5′-TTTGCTGATCCACATCTGCT-3′, *SOX17_forward* 5′-TTTGAACAGTATCTGCACTTCG-3′, *SOX17_reverse* 5′-TGCAGTAATATACCGCGGAG-3′, *MAP2_forward 5′-AGGTGGTAATGTCAAGATTGACAG-3′*, *MAP2_reverse* 5′-CAAAGTGGCAAGCTGAGGAG-3’.

#### 2.2.7 RNA isolation, library preparation, and sequencing

Total RNA was extracted from primary colorectal tumor biopsy samples, matched healthy biopsy samples obtained from non-tumor regions when applicable, and iIOs using PureZOL reagent (Bio-Rad) according to the manufacturer's instructions. Isolated RNA was used for transcriptomic profiling. Biopsy samples were sequenced on the MGI DNBSEQ-T7 platform; iIO samples were sequenced on the Illumina NextSeq 6000 platform using the Illumina RNA Library Prep Set (catalogue no. 20040525). Biopsy samples' RNA-seq libraries were prepared using the Watchmaker RNA Library Prep Kit with Polaris™ Depletion (Watchmaker Genomics), following the manufacturer's Library Construction Protocol. Sequencing was performed on the MGI DNBSEQ-T7 platform. Platform differences were assessed by PCA and sample-correlation diagnostics; the dominant axis of variation was biological context rather than sequencing platform.

#### 2.2.8 Read processing and alignment (FastQC, cutadapt, STAR 2-pass, GRCh38)

Raw sequencing reads were quality-checked with FastQC and adapter-trimmed with Cutadapt. Cleaned reads were aligned to the GRCh38 human reference genome with the STAR aligner in two-pass mode, and gene-level read counts were generated concurrently with alignment [31]. Ensembl gene identifiers were mapped to official HGNC gene symbols, and counts for duplicate symbols were summed.

#### 2.2.9 Differential expression analysis (paired PyDESeq2)

Differential expression was performed with DESeq2 [32] using a patient-paired design (∼ patient + condition) to control for inter-patient variation, with counts normalized by the median-of-ratios method; the analysis was implemented reproducibly in Python with PyDESeq2 [33]. The primary contrast compared primary tumor biopsies with iPSC-derived intestinal organoids (iIOs; reference level = iIO, such that a positive log2 fold-change denotes higher expression in biopsies). The same paired design was applied to the tumour-versus-matched-normal and matched-normal-versus-iIO contrasts. High-confidence differentially expressed genes (DEGs) were defined as Benjamini–Hochberg adjusted p < 0.05, absolute log2 fold-change > 1, and baseMean > 10, the last excluding low-abundance transcripts. Gene-level filtering was performed after alignment and quantification, on the STAR-generated count matrix and prior to differential expression analysis. Genes with at least 10 reads in at least six samples were retained. The six-sample threshold corresponds to the size of the smallest experimental group (n = 6 per group), ensuring that genes robustly expressed in at least one full group were kept while genes detected only sporadically were removed; this reduces noise from lowly expressed genes and stabilizes dispersion estimation and size-factor normalization in DESeq2.

#### 2.2.10 Unsupervised analysis and pathway enrichment

Counts were transformed with the variance-stabilizing transformation (VST) for unsupervised analyses, which limits the disproportionate influence of highly abundant transcripts. Principal component analysis was computed on VST values to evaluate group separation among primary tumor biopsies, matched normal biopsies and iIOs. Sample-level similarity was further assessed by Pearson correlation and Euclidean distance matrices, and group structure was visualized by hierarchical clustering of DEGs. Volcano and MA plots summarized the magnitude and significance of differential expression. Pathway activity was assessed by pre-ranked gene-set enrichment analysis (GSEA) [34], with genes ranked by sign(log2FC) × −log10(adjusted p) and tested against the MSigDB Hallmark collection [35]; pathways with a false-discovery rate < 0.05 were considered significant. For the functional-program summary, Hallmark pathways were grouped a priori into broad programs and the mean normalized enrichment score per program was reported. A leading-edge gene heatmap displayed genes that simultaneously (i) fell within the leading edge of their pathway, (ii) were significantly differentially expressed, and (iii) showed a fold-change direction concordant with their program. GSEA and single-sample GSEA were performed with gseapy [36].

#### 2.2.11 Curated signature scoring

For targeted interpretation, signatures were defined as explicit, fully documented gene lists (provided in full in Supplementary Table S1) spanning intestinal lineage specification, crypt–villus organization, intestinal identity, epithelial barrier integrity, extracellular-matrix remodeling, EMT/stromal contamination, metabolic programs, chromatin-associated regulation, and detoxification/defense pathways; a gene could contribute to more than one panel. Per-sample signature scores were computed as the mean of z-scored VST values across the member genes of each panel. Group differences were assessed by patient-paired t-tests with Benjamini–Hochberg correction, and scores were min–max scaled to [0,1] for radar visualization.

#### 2.2.12 Matched-normal and fetal/adult developmental analysis

To define which features of healthy intestinal epithelium are reacquired by organoids, DESeq2 results for the normal-biopsy-versus-organoid comparison were integrated across the CRC-iIO samples to identify consensus genes. Consensus genes that were higher in normal mucosa ("not reacquired") or higher in organoids ("retained") were tested for Hallmark over-representation (hypergeometric test, Benjamini–Hochberg corrected). Enrichment of the organoid-higher set for fetal epithelial genes was evaluated by Fisher's exact test. Fetal and adult epithelial signatures were derived from fetal and adult epithelial pseudobulk profiles of the Gut Cell Atlas [37]. The Human Gut Cell Atlas epithelial compartment comprised 142,113 cells across fetal, pediatric, and adult donors. Top150 genes by fetal/adult log2 fold change, excluding immunoglobulin and mitochondrial genes, were used as fetal and adult signature gene lists. All samples were scored by single-sample GSEA (rank-based, minimum set size 10) on log2-CPM-normalized values; rank-based scoring was chosen for robustness to cross-platform expression-scale differences.

#### 2.2.13 Crypt–surface zonation analysis

Curated colonic crypt-base (stem/proliferative) and surface (differentiated colonocyte) signatures were scored as the mean of z-scored expression. A zonation index (surface minus crypt score) positioned each sample along the crypt–surface axis, and groups were compared by patient-paired t-tests.

#### 2.2.14 Epithelial lineage scoring and NNLS deconvolution

Epithelial lineage representation was assessed by two complementary approaches. First, curated colonic lineage signatures (stem, transit-amplifying, mature colonocyte, goblet, enteroendocrine, tuft, BEST4+ and deep-crypt secretory) were scored by single-sample GSEA [38]. Second, absolute lineage proportions were estimated by reference-based deconvolution: a signature matrix was constructed from Gut Cell Atlas epithelial pseudobulk [37] (sub-types collapsed to major lineages and CPM-normalized, with cell-type-specific marker genes selected by specificity), and non-negative least squares estimated lineage weights per bulk sample, normalized to proportions. Because this reference is epithelial-only, biopsy proportions reflect the epithelial-signal composition rather than true cell-type fractions; stromal and immune content was quantified separately by MCP-counter. The uncertainty inherent to transcript-based deconvolution, including reference incompleteness and confounding by non-epithelial signal in biopsies, is acknowledged as a limitation of this approach.

#### 2.2.15 Metabolic module analysis

Ten curated metabolic modules and a proliferation module were scored per sample by single-sample GSEA. A Warburg index (glycolysis minus oxidative phosphorylation) summarized the glycolytic-versus-oxidative balance. The coupling between anabolic metabolism and proliferation was quantified as the Pearson correlation between a composite biosynthetic score and the proliferation score across samples, computed both overall and per module.

#### 2.2.16 Consensus molecular subtype (CMS) classification

Consensus molecular subtypes were assigned to the six primary tumor biopsies by nearest-template prediction using the CMScaller framework [39], with subtype templates applied to log-transformed expression and significance assessed by permutation with Benjamini–Hochberg correction. Because the CMS framework was derived from bulk tumors and incorporates stromal/immune signals absent from epithelial organoids, classification was restricted to the biopsies and is reported as a descriptive cohort characterization (Fig. S3 A–B).

#### 2.2.17 Malignancy-axis analysis

To test whether iIOs carry a malignant transcriptional tendency, a malignancy axis was derived from the cohort's own tumor-versus-normal differential expression: genes significantly higher in tumor ("Tumor-up") and higher in normal mucosa ("Normal-up") were each scored per sample by single-sample GSEA [38] on log2 CPM values, and a malignancy index was computed as Tumor-up minus Normal-up. Group differences were assessed by two-sided patient-paired t-tests (Fig. S3D).

#### 2.2.18 Immune and stromal composition (MCP-counter)

Immune and stromal content was estimated with MCP-counter marker genes [40]: for each population, the score was the mean log2-CPM of its marker genes, and aggregate immune and stromal scores were compared across groups (tumor, normal, iIO) by patient-paired t-tests with BH correction.

### 2.3 Quantification and statistical analysis

Summary statistics are reported as mean ± SD. Group comparisons used two-sided patient-paired t-tests; categorical enrichment used Fisher's exact test, and correlations used Pearson's r. The patient-paired *t*-test was chosen as the primary inferential framework because all comparisons involve the same six patients contributing matched samples across conditions (Normal, Tumor, iIO), rendering observations within each patient non-independent. This paired design is statistically equivalent to a linear mixed model with patient as a random intercept, and it directly controls for inter-patient variation in baseline expression, the dominant source of variance in this cohort (PC1, 63% of total variance). The paired *t*-test assumes (i) that differences between paired observations are continuously distributed and (ii) that these within-patient differences are approximately normally distributed. With n = 6 pairs, formal normality tests (e.g. Shapiro-Wilk) have low power and were therefore not used as a gating criterion; instead, all primary comparisons were independently re-tested with the Wilcoxon signed-ranked test, which carries no normality assumption. Paired *t*-test and Wilcoxon results were concordant for all but two comparisons (crypt-surface zonation iIO vs Normal, and glutaminolysis iIO vs Normal), where the Wilcoxon p-value exceeded the BH-corrected threshold. The discordance in these two cases reflects the known low resolution of the Wilcoxon test at n = 6 (minimum achievable two-sided p = 0.031), not a reversal of direction. No outliers were removed from any comparison. Full test results, including n pairs, raw paired *t*-test p, Wilcoxon signed-rank p, Cohen's dz, 95% confidence interval, BH-adjusted p for both tests and concordance, are provided in Supp. Table S3.Multiple testing was controlled by the Benjamini–Hochberg procedure. Analyses were performed in Python 3.12 with PyDESeq2 (v0.5.4), gseapy (v1.2.1), pandas (v2.3.3), numpy (v2.4.4), scipy (v1.17.1) and statsmodels (v0.14.6). All processing scripts, with environment specification and run order, are provided as Data S1. Figures were generated in Python (matplotlib) and included PCA, volcano and MA plots, heatmaps, radar plots, bar and bubble plots. Directionality was indicated with log2 fold-change axes and contextual group labels (e.g. “higher in biopsy", "higher in organoid", "higher in healthy mucosa"). Statistical details, including n, the test used, and the definition of significance, are reported in the figure legends and Results.

## 3 Results

### 3.1 Generation and characterization of iPSC-derived intestinal organoids

Directed differentiation of iPSCs toward intestinal organoids was validated by early lineage specification and organoid marker analysis. Flow cytometry showed efficient induction of definitive endoderm-associated markers, with high CXCR4 positivity and prominent CD117 expression, while PDGFRα expression remained low, indicating preferential endodermal specification (Fig. 1A–C). During subsequent differentiation, immunofluorescence imaging showed increased CDX2 expression from day 3 to day 9, supporting progression toward hindgut/intestinal specification, and three-dimensional culture generated spheroid/organoid-like epithelial structures (Fig. 1B–D). Established organoids expressed intestinal epithelial, progenitor, and differentiated lineage markers, including Chromogranin A, KLF5, FOXA2, Villin, Lysozyme, LGR5, and CDX2 (Fig. 1E). These results confirm successful generation of iPSC-derived intestinal organoids with preserved intestinal epithelial identity.

**Fig. 1:**
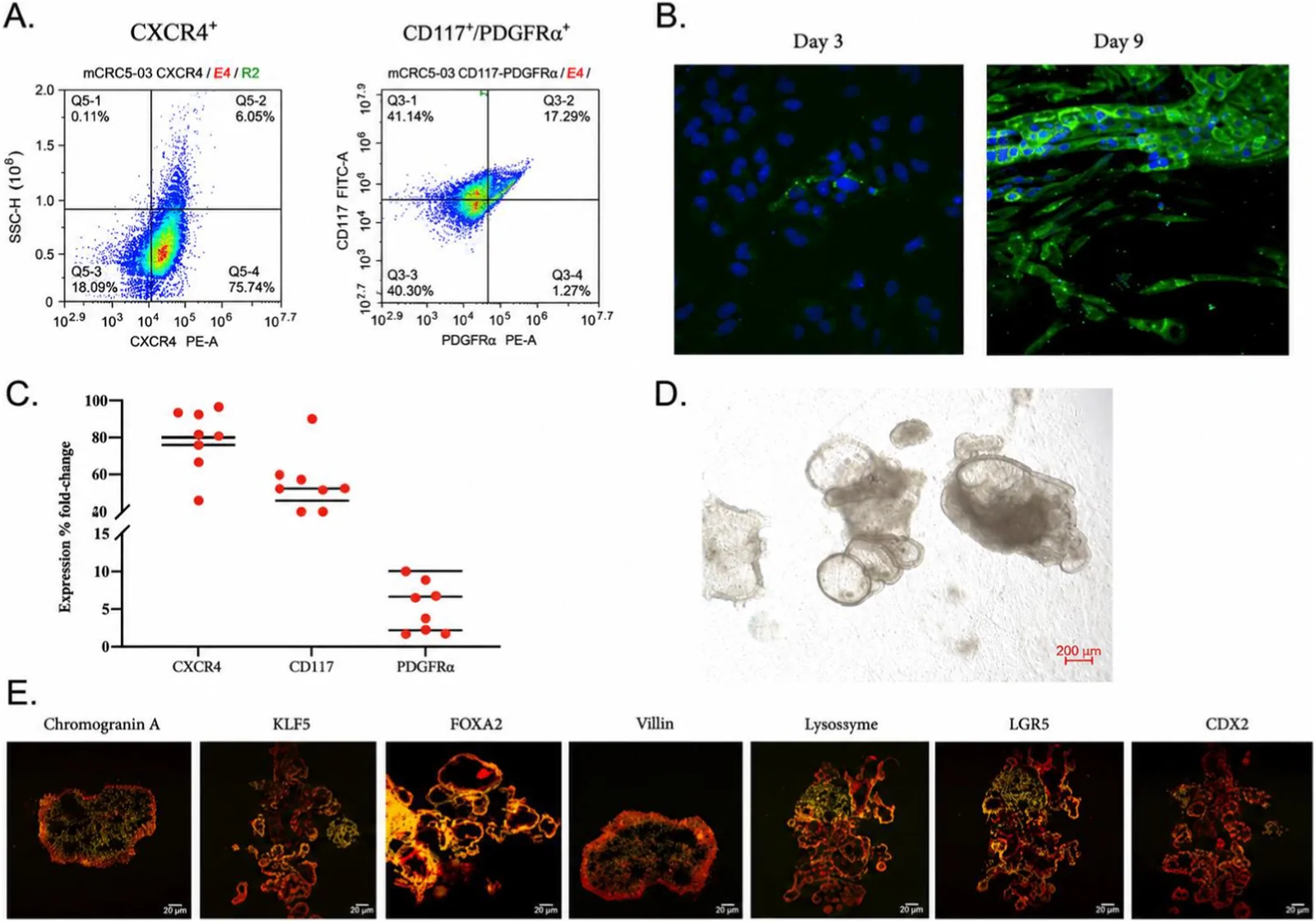
**Generation and characterization of iPSC-derived intestinal organoids.** (**A)** Representative flow cytometry plots showing expression of definitive endoderm-associated markers CXCR4 and CD117, together with PDGFRα. (**B)** Representative immunofluorescence images showing lineage-associated marker expression at day 3 and day 9 of differentiation. Green indicates CDX2, and blue indicates DAPI nuclear staining. (**C)** Quantification of CXCR4, CD117, and PDGFRα expression levels during early differentiation. Data are shown as individual biological replicates with mean values indicated by horizontal bars. (**D)** Representative bright-field image of iPSC-derived intestinal organoid/spheroid structures generated during three-dimensional culture. Scale bar: 200 μm. **(E)** Immunofluorescence characterization of established iIOs showing expression of Chromogranin A, KLF5, FOXA2, Villin, Lysozyme, LGR5, and CDX2. Nuclei are counterstained with DAPI where applicable [30].

### 3.2 iIOs diverge from primary colorectal tissue more than tumors differ from matched normal mucosa

To define the global transcriptional relationship between primary colorectal cancer tissues and a developmentally organized epithelial reference, we first compared primary CRC biopsy transcriptomes with iIOs. Principal component analysis revealed a clear separation between biopsy and iIO samples, with PC1 explaining 63.0% of the total variance, indicating that the dominant source of transcriptional variation was the biological context of the samples. Biopsy samples displayed broader dispersion, consistent with the heterogeneous cellular and microenvironmental composition of primary tumor tissues, whereas iIO samples formed a more compact cluster (Fig. 2A). Differential expression analysis further confirmed extensive transcriptomic divergence between the two systems (Fig. 2B). Additionally, paired differential expression showed that iIOs diverge from intestinal tissue far more than tumor and normal tissue differ from each other. The tumor-versus-matched-normal comparison yielded 732 differentially expressed genes, whereas organoid-versus-tumor and organoid-versus-normal comparisons yielded 10,476 and 8692 genes, respectively (Fig. S1C). CRC biopsies and iIOs therefore occupy clearly distinct transcriptional states, reflecting the contrast between complex tumor tissue and an epithelial organoid reference. Gene-set enrichment attributed this divergence to proliferative programs (MYC, E2F, G2M) in iIOs versus immune and inflammatory signaling in tissue (Fig. 3).

**Fig. 2:**
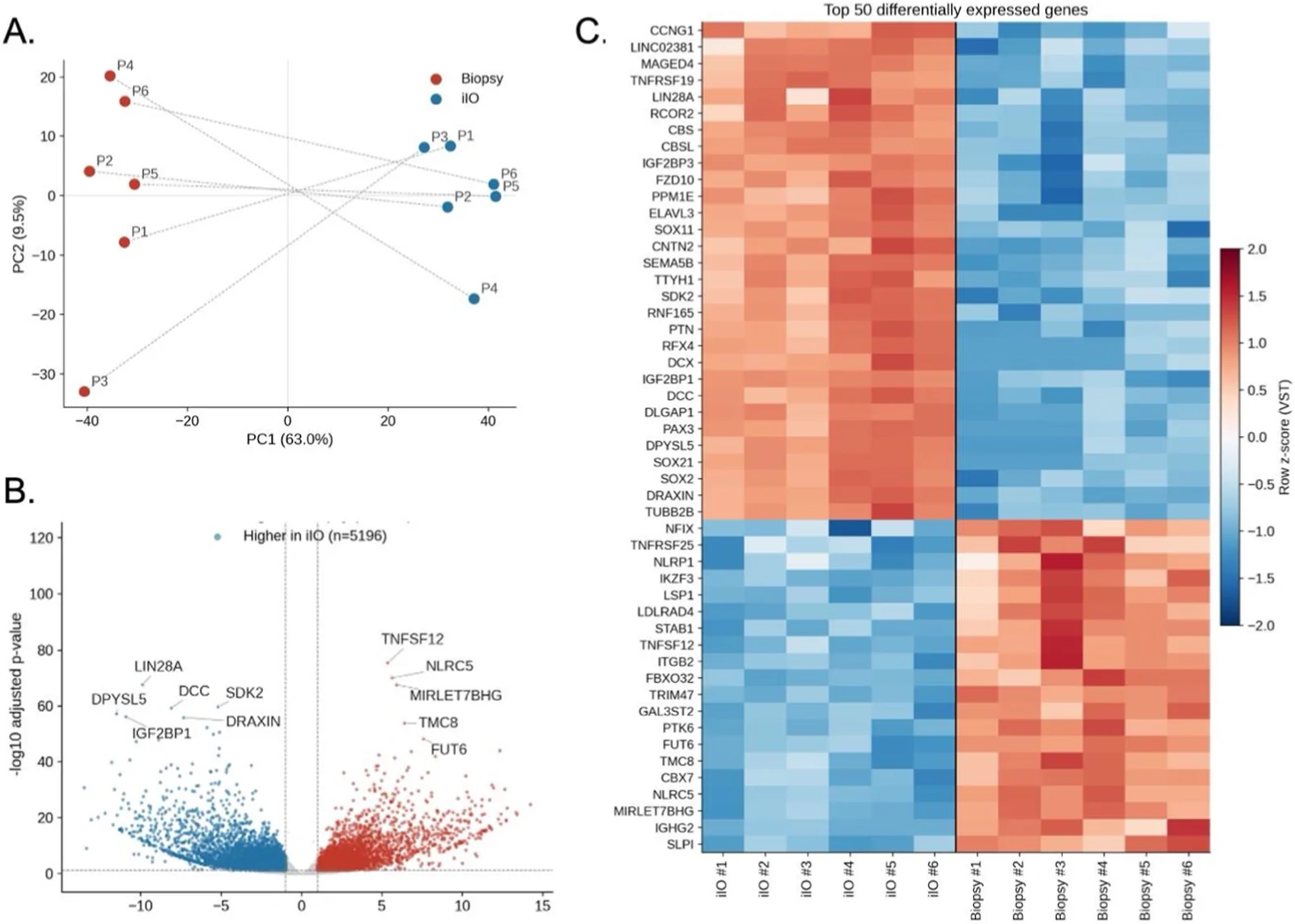
**iIOs diverge from primary colorectal tissue more than tumors differ from matched normal mucosa (A)** Principal component analysis showing separation of primary CRC biopsy samples and iIOs along PC1, which explains 63.0% of the total variance. Dashed lines connect matched or corresponding sample labels where applicable. **(B)** MA plot showing differentially expressed genes between biopsy and iIO samples. Red dots indicate genes upregulated in biopsies, whereas blue dots indicate genes upregulated in iIOs. **(C)** Volcano plot displaying the magnitude and significance of differential gene expression between biopsies and iIOs. Selected highly significant genes are labeled. Red indicates genes higher in biopsies, and blue indicates genes higher in iIOs.

**Fig. 3:**
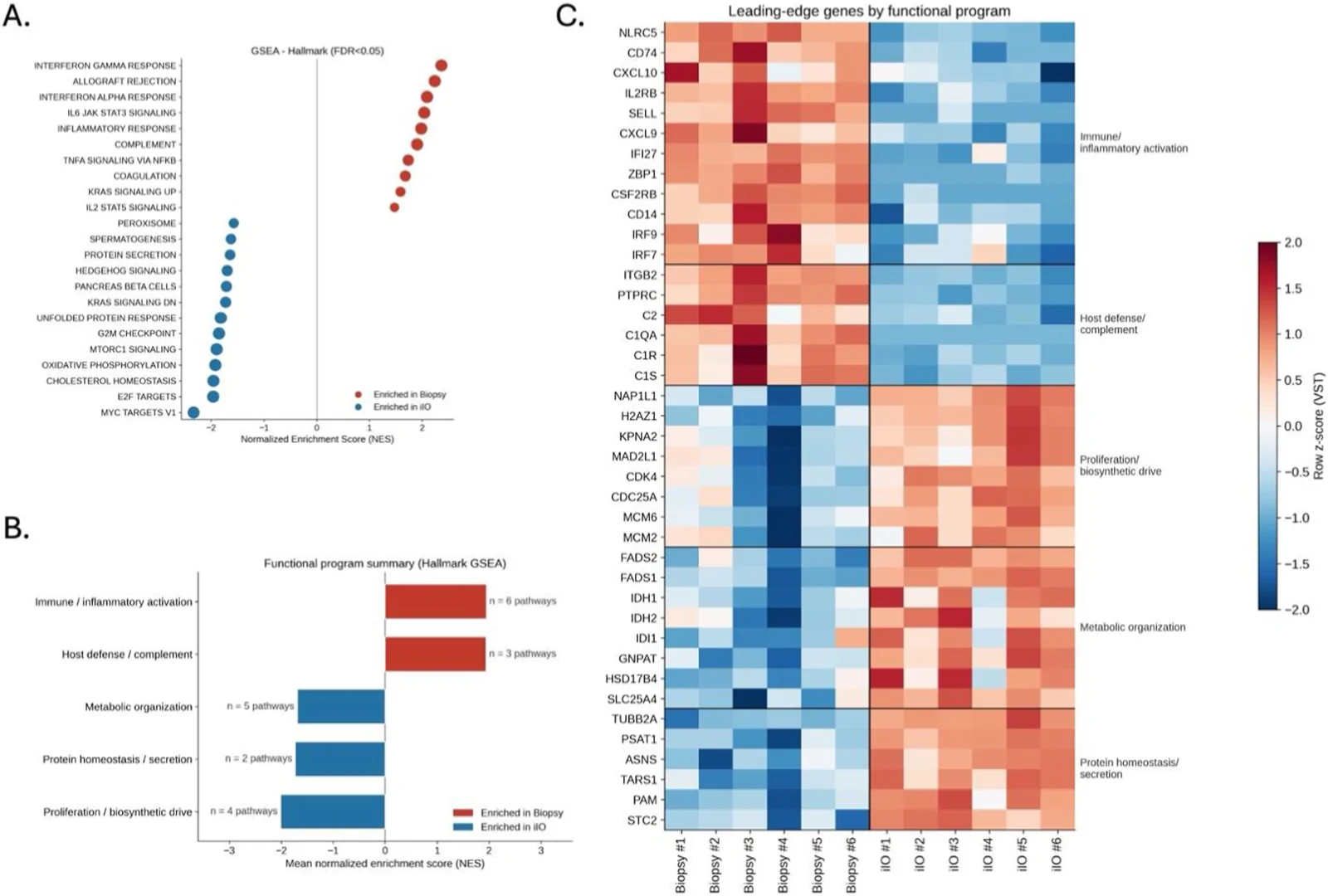
**Biopsies are dominated by immune–stromal programs, whereas iIOs are enriched for proliferative and biosynthetic programs (A)** Hallmark gene set enrichment analysis of the biopsy vs iIO. Red dots indicate biopsy-enriched gene sets and blue dots indicate iIO-enriched gene sets. Only significantly enriched gene sets are shown. **(B)** Functional program summary of significantly enriched Hallmark gene sets. Related gene sets were grouped into major biological programs, and the mean normalized enrichment score was calculated for each program. **(C)** Heatmap of representative leading-edge genes selected from major biopsy- and iIO-enriched Hallmark programs. Expression values are row-scaled across samples.

### 3.3 Biopsies are dominated by immune–stromal programs, whereas iIOs are enriched for proliferative and biosynthetic programs

To functionally interpret the broad transcriptional divergence between biopsies and iIOs, we performed gene set enrichment analysis (GSEA) using the Hallmark gene set collection [35]. Hallmark GSEA revealed a clear functional separation between biopsy and iIO samples (Fig. 3). Biopsy samples were strongly enriched for immune and inflammatory programs, whereas iIO samples were enriched for biosynthetic, metabolic, proliferative, and protein homeostasis-associated programs (Fig. 3A). Consistent with the Hallmark analysis, KEGG and Reactome GSEA further supported the same directional pattern of functional divergence. KEGG analysis showed enrichment of immune-related pathways in biopsy samples, while Reactome analysis highlighted immunoregulatory interactions, complement activation, and Fc receptor-associated immune pathways. Conversely, iIO-enriched KEGG and Reactome terms were predominantly related to ribosome, translation, RNA processing, and protein homeostasis, further supporting the biosynthetic character of the organoid state (Fig. S2A and B). To summarize these pathway-level differences at the program level, we grouped significantly enriched Hallmark gene sets into major biological categories and calculated the mean normalized enrichment score for each program. This analysis underscored the dominant enrichment of immune/inflammatory activation and host defense/complement-associated programs in biopsy samples, whereas iIOs showed higher enrichment of proliferation/biosynthetic drive, metabolic organization, and protein homeostasis/secretion programs (Fig. 3B). These results indicate that primary CRC biopsies are transcriptionally dominated by immune and tissue-level inflammatory signaling, whereas iIOs retain a metabolically active and biosynthetically organized epithelial state.

To further visualize the gene-level contribution of these enriched pathways, we generated a heatmap of representative leading-edge genes selected from the major biopsy- and iIO-enriched Hallmark programs (Fig. 3C). This analysis showed coordinated enrichment of immune, inflammatory, antigen presentation, leukocyte-associated, and complement-related genes in biopsies, whereas iIOs showed higher expression of genes associated with proliferation, metabolic organization, and protein homeostasis. At the gene level, the divergence between biopsy and iIOs separates immune/stromal-rich tumor tissue from an epithelial organoid reference dominated by metabolic and biosynthetic activity.

We also performed signature score analysis of predefined intestinal lineage, metabolic, stromal, EMT/mesenchymal, and immune programs. iIOs showed higher scores for several epithelial lineage-associated and metabolic signatures, including absorptive enterocyte, Paneth, enteroendocrine, Notch-associated, lipid synthesis, OXPHOS, and TCA cycle programs. By contrast, biopsy samples showed higher stromal/fibroblast and immune infiltration scores, supporting the presence of tissue-level microenvironmental components (Fig. S2C). Consistently, cell population deconvolution further showed higher immune and stromal content in primary tumor and normal tissue samples compared with iIOs, supporting the interpretation that biopsy-enriched inflammatory and stromal programs reflect the multicellular microenvironmental composition of primary tissues rather than epithelial-intrinsic differences alone (Fig. S3C). CMS classification is reported descriptively for the six tumor biopsies and is not used to support any general or subtype-level claim This multicellular composition is directly supported by MCP-counter quantification, which confirmed significantly elevated immune and stromal scores in biopsies relative to iIOs, and by NNLS deconvolution (Fig. 6D), which showed that the dominant lineage signal in biopsies includes non-epithelial contributions absent from organoids. Accordingly, immune and inflammatory gene set enrichment in biopsies should not be interpreted as tumor-intrinsic epithelial programs.

### 3.4 iIOs fail to reacquire adult mucosal programs and retain a fetal-like epithelial state

To define which features of the healthy intestinal epithelium are reacquired by organoids, matched normal-biopsy-versus-organoid comparisons were analyzed. The iIOs were highly concordant (log2FC Pearson r = 0.74; Fig. 4A), yielding 1705 genes consistently higher in normal mucosa and 1251 consistently higher in organoids. Genes lost in organoids, defined as genes higher in normal mucosa, were enriched for immune, inflammatory and stromal programs reflecting microenvironmental compartments absent from epithelial organoids (Fig. 4B). Conversely, genes gained in organoids were enriched for MYC targets, E2F targets, MTORC1 signaling, G2M checkpoint, unfolded protein response, and cholesterol homeostasis (Fig. 4C). This organoid-gained program was, in turn, strongly enriched for fetal epithelial genes (Fisher OR = 9.8, p = 2 × 10^−21^; e.g. AFP, NAP1L1, NPM1, HMGA1), prompting direct assessment of developmental state. Scoring all samples on Gut Cell Atlas fetal and adult epithelial signatures placed iIOs along the diagonal (fetal ≈ adult), whereas normal and tumor tissue lay below it (adult > fetal) (Fig. 4D). The resulting developmental index (fetal − adult) was significantly higher in iIOs (≈0.00) than in normal (−0.32) or tumor (−0.37) tissue (normal vs iIO p = 0.004; tumor vs iIO p = 0.0005; Fig. 4E). iIOs thus reacquire neither the tissue-context (immune/stromal) nor the mature-differentiation features of healthy mucosa, and instead retain a proliferative, fetal-like epithelial program. To independently validate the RNA-seq-derived fetal and progenitor-biased identity of iIOs, qRT-PCR was performed on all six patient-matched iPSC-iIO pairs. Relative to parental iPSCs, iIOs expressed AFP, CDX2, and LGR5 at 121-, 98-, and 386-fold higher levels, respectively (Fig. 4F). The validation confirms acquisition of fetal intestinal epithelial identity and crypt/progenitor program activation upon directed differentiation.

**Fig. 4:**
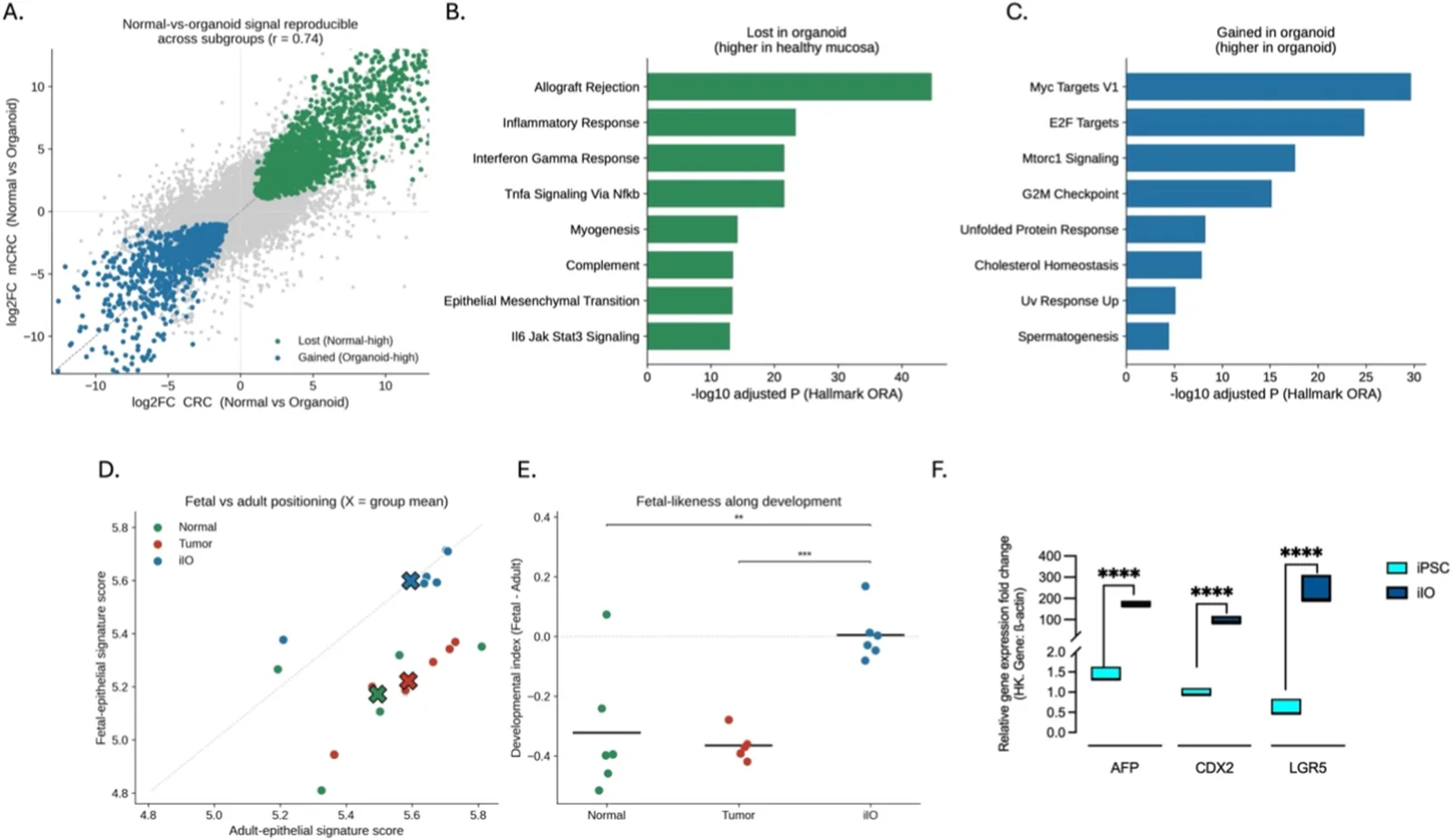
**iIOs fail to reacquire adult mucosal programs and retain a fetal-like epithelial state. (A)** Scatter plot comparing normal mucosa-versus-organoid log2 fold-change signatures across independent subgroup contexts. Each dot represents a gene. Green dots indicate genes reproducibly higher in normal mucosa and reduced in organoids, referred to as lost in organoid. Blue dots indicate genes reproducibly higher in organoids, referred to as gained in organoid. Gray dots indicate genes not classified in either reproducible category. The Pearson correlation coefficient is shown in the panel title. **(B)** Hallmark over-representation analysis of genes lost in organoids, defined as genes with higher expression in normal mucosa. Bar length represents –log10 adjusted P value. **(C)** Hallmark over-representation analysis of genes gained in organoids, defined as genes with higher expression in organoids. Bar length represents –log10 adjusted p-value. **(D)** Fetal versus adult epithelial signature positioning of normal tissue, tumor biopsy, and iIO samples. Individual samples are shown as dots, and group means are indicated by larger crossed markers. **(E)** Developmental index calculated as the relative balance between fetal-like and adult-like epithelial signature scores. Higher values indicate stronger fetal-like epithelial identity. **(F)** qRT-PCR validation of fetal intestinal identity and crypt/progenitor markers in iIOs relative to their parental iPSCs (n = 6). Expression values are shown as mean ± SD, normalized to β-actin. Statistical comparison by two-sided paired *t*-test, ****p < 0.0001.

### 3.5 iIOs occupy a crypt-base/proliferative position on the crypt–surface axis

Having established the broad functional divergence between CRC biopsies and iIOs, we next examined whether iIOs retained intestinal epithelial organization along the crypt–surface axis. Projection of normal tissue, tumor biopsy, and iIO samples using crypt-base/stem-proliferative and surface-differentiation signature scores showed that iIOs occupied a crypt/proliferative-biased epithelial state, with relatively lower surface differentiation scores compared with normal tissue samples (Fig. 5A). Consistently, the crypt-surface zonation index (surface minus crypt signature) ordered the groups as normal mucosa (+0.79) > tumor (+0.05) > iIO (−0.84), with a significant difference between normal tissue and organoids (p = 0.013, Fig. 5B). iIOs were biased toward the crypt/proliferative pole, indicating that the crypt-villus axis is partially, rather than fully, reconstituted. Heatmap analysis of representative crypt-base and surface marker genes further supported this pattern (Fig. 5C). iIO samples showed coordinated expression of crypt-base and proliferation-associated genes, including MKI67, PCNA, TOP2A, BIRC5, CCNB1, CDK1, FOXM1, and MCM2, whereas surface-associated differentiation markers were more variably represented across primary normal and tumor tissues and were less consistently enriched in iIOs. We further assessed tissue architecture, epithelial barrier, matrix remodeling, and defense-associated signatures. This analysis showed that iIOs retained structural and epithelial-associated tissue features, whereas biopsy samples showed relatively higher matrix remodeling activity, consistent with the tissue-level complexity of primary samples (Fig. 5D). Overall, iIOs retain intestinal epithelial identity but remain biased toward the crypt-base/proliferative compartment, without fully reproducing the surface-differentiated organization of primary tissue.

**Fig. 5:**
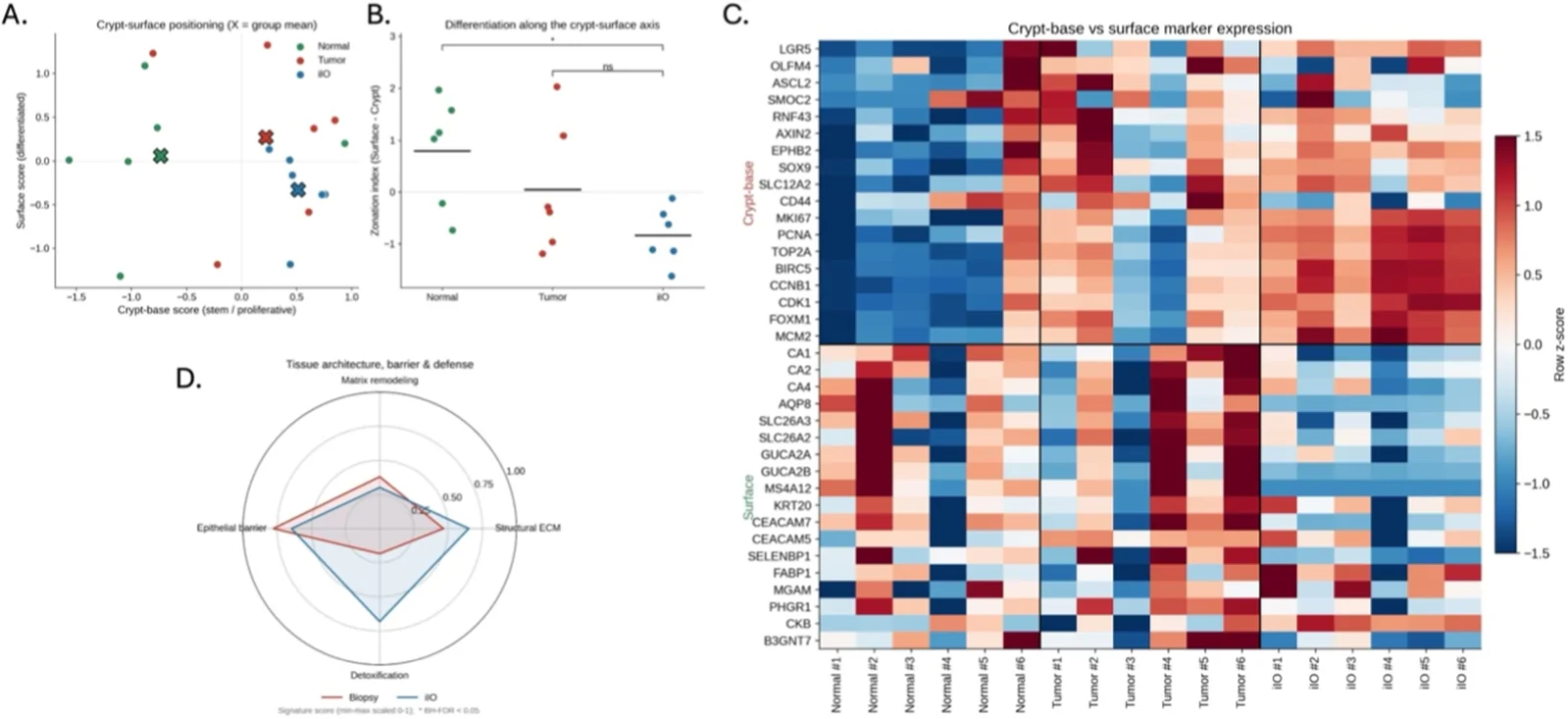
**iIOs occupy a crypt-base/proliferative position on the crypt–surface axis (A)** Crypt–surface positioning analysis based on crypt-base/stem-proliferative and surface-differentiation signature scores in normal tissue, tumor biopsy, and iIO samples. Individual samples are shown as dots, and group means are indicated by larger crossed markers. **(B)** Zonation index calculated as the relative balance between surface-associated and crypt-base-associated programs. Higher values indicate stronger surface-differentiation relative to crypt-base/proliferative identity. **(C)** Heatmap showing row-scaled expression of representative crypt-base/proliferative and surface-differentiation marker genes across normal tissue, tumor biopsy, and iIO samples. Genes are grouped according to crypt-base and surface-associated epithelial programs. **(D)** Radar plot showing min–max normalized tissue architecture, epithelial barrier, matrix remodeling, and defense-associated signature scores in biopsy and iIO samples. Asterisks indicate significantly different signatures after Benjamini–Hochberg correction.

**Fig. 6:**
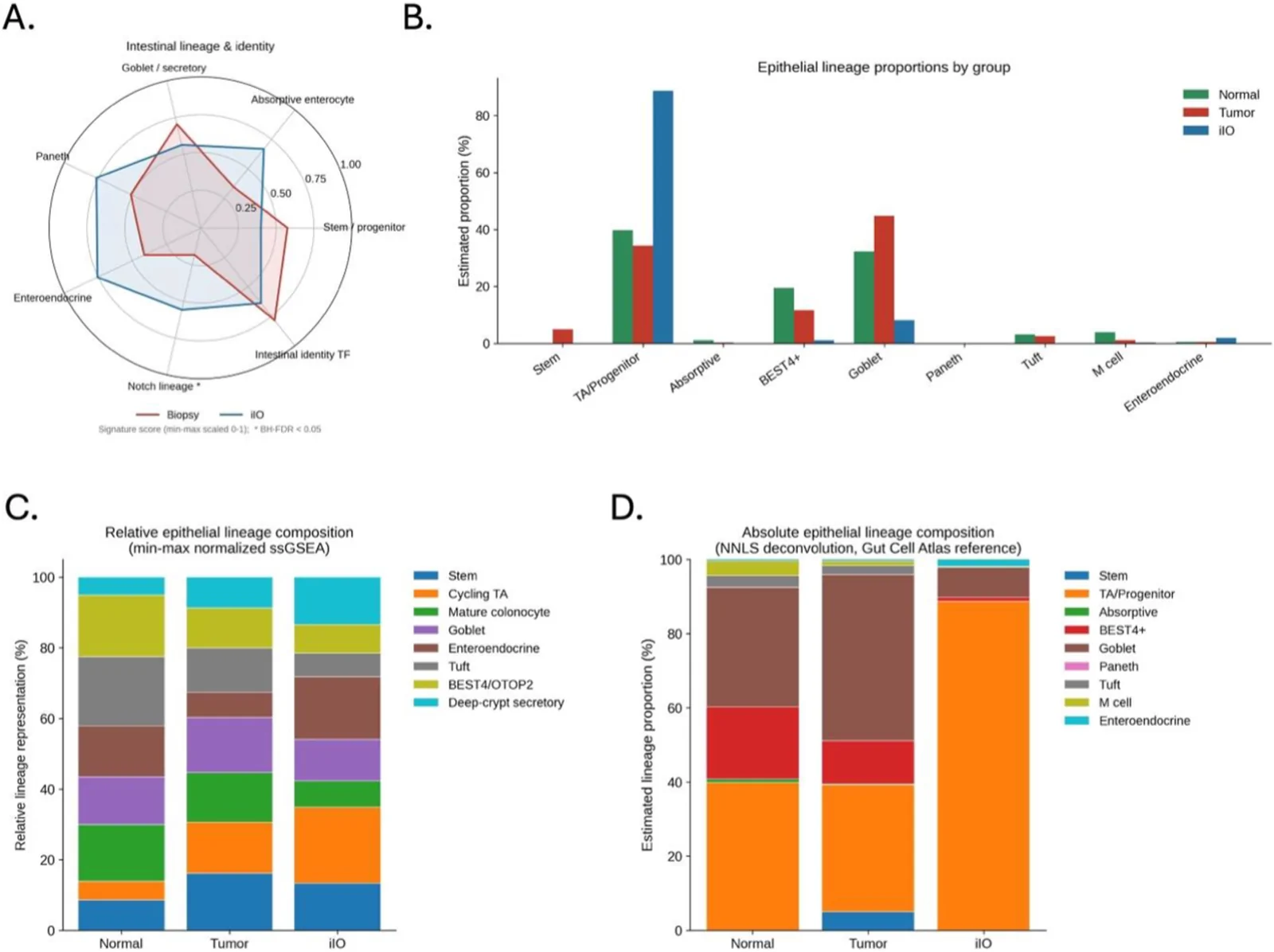
**iIOs are biased toward transit-amplifying/progenitor lineages and depleted of differentiated cell types (A)** Radar plot showing min–max normalized intestinal lineage and identity signature scores in biopsy and iIO samples. Asterisks indicate significantly different signatures after Benjamini–Hochberg correction. **(B)** Group-wise estimated epithelial lineage proportions across normal tissue, tumor biopsy, and iIO samples. Bars show the estimated contribution of each epithelial lineage-associated program per group. **(C)** Relative epithelial lineage composition based on min–max normalized ssGSEA scores for predefined intestinal epithelial lineage signatures. Stacked bars show the relative representation of each epithelial lineage-associated program within normal tissue, tumor biopsy, and iIO groups. **(D)** Absolute epithelial lineage composition estimated by non-negative least squares deconvolution using Gut Cell Atlas epithelial lineage references. Stacked bars show the estimated proportional contribution of each epithelial lineage across normal tissue, tumor biopsy, and iIO groups.

### 3.6 iIOs are biased toward transit-amplifying/progenitor lineages and depleted of differentiated cell types

We next assessed epithelial lineage composition to further define the epithelial state represented by iIOs. Signature score analysis indicated that iIOs retained multiple intestinal lineage-associated programs, including absorptive enterocyte, Paneth, enteroendocrine, and Notch-associated signatures, while biopsy samples showed relatively higher stem (LGR5), goblet/secretory, and intestinal identity transcription factor-associated scores (Fig. 6A). These signature scores reflect the transcriptional activity of lineage-associated programs rather than the proportion of each cell type; accordingly, the retention of enterocyte- and secretory-associated programs in iIOs is not equivalent to a mature-lineage composition, which we assessed separately below. Group-level lineage proportion analysis further showed that iIOs were strongly dominated by transit-amplifying/progenitor-like (cycling TA) epithelial states, whereas normal and tumor tissues retained greater contributions from differentiated epithelial lineages such as goblet and BEST4-positive populations (Fig. 6B). At the level of lineage composition, ssGSEA of curated colonic lineage signatures showed iIOs enriched for cycling transit-amplifying cells (p = 0.010 vs normal) and depleted of mature colonocytes (p = 0.008), BEST4+ cells (p = 0.001) and tuft cells (p = 0.027), with a relative excess of enteroendocrine cells (p = 0.023, Fig. 6C). Absolute epithelial lineage deconvolution using Gut Cell Atlas reference signatures independently confirmed the marked transit-amplifying/progenitor bias of iIOs (Fig. 6D). iIOs therefore preserve intestinal epithelial lineage identity but remain a progenitor-biased state with limited differentiated lineage diversity.

### 3.7 Elevated iIO metabolism reflects proliferation-coupled anabolism

To evaluate whether iIOs adopt a distinct metabolic state, curated metabolic programs were scored per sample. Relative to biopsies, iIOs showed broadly elevated metabolic signatures across lipid synthesis (BH-FDR < 0.05), glycolysis, the TCA cycle and oxidative phosphorylation (Fig. 7A). Module-level scoring confirmed a coordinated up-regulation of biosynthetic programs in iIOs together with glycolysis and oxidative phosphorylation, whereas fatty-acid oxidation was the notable exception (Fig. 7B). This elevation did not reflect a glycolytic (Warburg-type) shift: the glycolysis-minus-OXPHOS index was uniformly low and statistically indistinguishable between normal mucosa, tumor and iIOs (Fig. 7C). Instead, metabolic activity closely tracked proliferation: a composite anabolic/biosynthetic score correlated strongly with the proliferation score across all samples (Pearson r = 0.89, p = 8.9 × 10^−7^; Fig. 7D). Per-module coupling was strongest for nucleotide synthesis (r = 0.96), the pentose-phosphate pathway (r = 0.92), glutaminolysis (r = 0.83), cholesterol/mevalonate (r = 0.80) and glycolysis (r = 0.79), with the catabolic fatty-acid-oxidation module anti-correlated (r = −0.46) (Fig. 7E). At the transcript level, this pattern is consistent with proliferation-driven anabolism supporting biomass production rather than a malignancy-like metabolic switch. However, as these inferences are based on gene expression signatures, functional metabolic measurements such as isotope tracing, oxygen consumption or extracellular flux assays would be required to confirm the directionality and magnitude of actual metabolic fluxes.

**Fig. 7:**
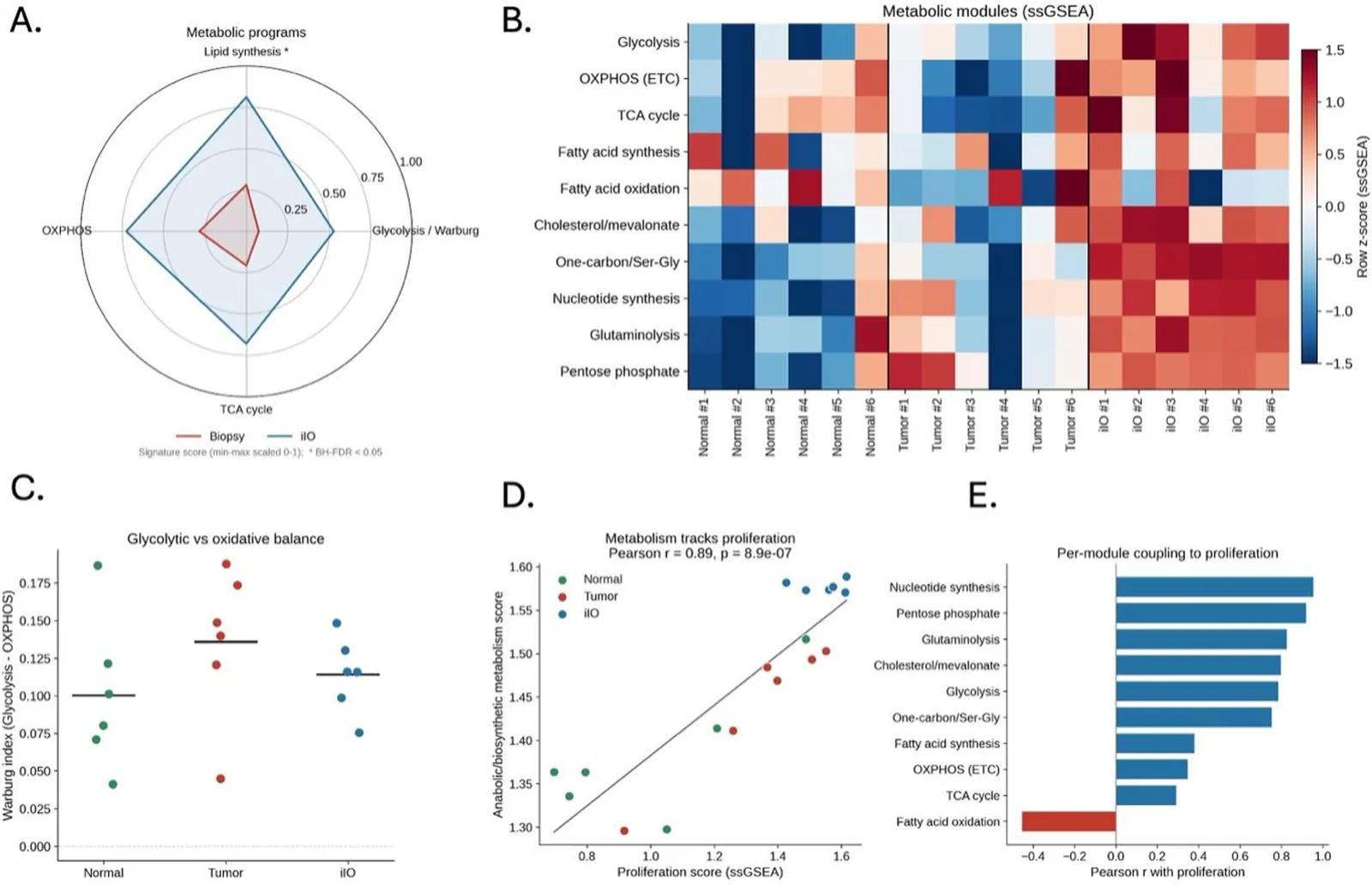
**Elevated iIO metabolic signatures are consistent with proliferation-coupled anabolism (A)** Radar plot showing min–max normalized metabolic program signature scores in biopsy and iIO samples. Asterisks indicate significantly different signatures after Benjamini–Hochberg correction. **(B)** Heatmap of metabolic module activity calculated by single-sample gene set enrichment analysis across normal tissue, tumor biopsy, and iIO samples. Values are shown as row-scaled ssGSEA scores. **(C)** Glycolytic-versus-oxidative balance index calculated as the relative balance between glycolysis and OXPHOS module scores. Individual samples are shown as dots, and group means are indicated by horizontal bars. **(D)** Correlation between anabolic/biosynthetic metabolism score and proliferation score across samples. Each dot represents an individual sample, and the fitted regression line is shown. **(E)** Module-level correlation analysis showing the association between individual metabolic module scores and proliferation score. Bars represent Pearson correlation coefficients.

## 4 Discussion

Within the scope of this study, the enriched pathways, epithelial lineage compositions, tissue organization, and metabolic states of iIOs were examined within a transcriptomic framework in comparison with the CRC biopsies of the patients from whom they were derived and their matched healthy intestinal biopsies. The findings demonstrated that iIOs possess intestinal epithelial organization; however, compared with primary tissue, they exhibit an immature, highly proliferative, and fetal-like character. Another critical point at which iIOs differ from primary biopsies is that, although they are derived from iPSCs generated from CRC patients, their dominant transcriptional profile reflects developmental maturation stages rather than malignant transformation, and this directly affects how iIOs should be evaluated when used as a model.

When differential gene expression differences between the groups were evaluated, the fact that the number of genes changing between iIOs and both tumor and normal tissue biopsies was more than 10-fold higher than the number of genes changing between tumor and matched normal tissue biopsies demonstrated the magnitude of the divergence (Fig. S1C). When this divergence was examined in more detail, findings were obtained indicating that the reason for this separation was not the malignant nature of the tumor biopsies, but rather the fact that iIOs represent a purer and fetal-like epithelial intestinal model. It was known that, independent of their malignancy status, primary tissue biopsies contain different cell types such as immune cells and endothelial cells in addition to epithelial structures; in this study, iIOs were shown to be intestinal epithelial models obtained as a result of directed differentiation [41]. It is important to note that the immune, stromal, and inflammatory transcriptional dominance observed in biopsies reflects tissue-level microenvironment complexity rather than tumor-intrinsic epithelial programming. Because primary biopsies contain immune cells, cancer-associated fibroblasts, endothelial cells, and extracellular matrix components alongside neoplastic epithelium, their transcriptomic profiles capture the full multicellular ecosystem of the tumor microenvironment. This distinction is central to interpreting the biopsy-iIO contrast: the enrichment of immune and inflammatory programs in biopsies does not imply that these are properties of the CRC epithelium itself. The findings obtained from the pathway enrichment analyses supported this observation and showed that iIOs were enriched in proliferative and biosynthetic pathways (Fig. 3, Fig. S2). The subsequent deconvolution analysis confirmed that, as expected, iIOs showed markedly lower immune and stromal content (Fig. S3C).

Particularly when normal tissue biopsies and iIOs were compared, it was observed that the gene groups significantly increased in iIOs were especially enriched in proliferative programs and pathways (Fig. 4C), and fetal epithelial markers were enriched in iIOs (Fisher OR = 9.8). The findings indicated that the iIOs studied did not acquire adult tissue organization but instead exhibited a developmentally fetal-like epithelial organization. Studies in the literature report that PSC-derived intestinal models resemble the fetal intestine and can progress toward maturation through approaches such as transplantation or co-culture [42], [43], [44], [45]. Importantly, the reported maturation status of PSC-derived intestinal and colonic organoids is protocol-dependent and heterogeneous across studies: standard directed-differentiation protocols typically yield fetal-like epithelium, whereas extended culture, tuning of BMP and WNT signaling, or in vivo transplantation can drive more adult-like maturation [42], [45], [46]. Our findings therefore characterize iIOs generated under one widely used protocol and assessed at a single time point (day 120), and the degree of fetal- and progenitor-biased identity we described may differ in organoids produces under maturation-optimized conditions. This heterogeneity reinforces our central point that iIOs should be benchmarked against, rather than assumed to represent, the tissue state they are intended to model [47].

Transcriptomic lineage analyses allowed us to further detail the epithelial identity of iIOs. Crypt-surface zonation analyses showed that iIOs were biased toward the crypt/proliferative pole; our reference-based deconvolution predicted that the dominant composition of iIOs consisted of transit-amplifying/progenitor cells and was depleted in mature colonocyte, goblet, BEST4+, and tuft lineages. In the general framework, these results suggest that iIOs can accurately reconstitute the proliferative progenitor compartment of the crypt as the dominant population, but have not yet restored the terminally differentiated and specialized lineages of the mature mucosal structure [48]. At this point, it should be particularly noted that the transcriptomic data of the samples used in this study are based on bulk RNA-seq. Based on the quality and depth allowed by our RNA-seq data, an estimated observation was made by performing Gut Cell Atlas- and published dataset-based deconvolution analyses.

After observing in our enrichment analyses that proliferative programs and their associated pathways were particularly enriched, we directly tested whether the proliferative state of iIOs was associated with malignancy by projecting all samples onto a malignancy axis derived from the differentially expressed genes between tumor and normal tissue biopsies obtained from the same patients from whom the iIOs were derived. On this axis, as expected, tumors scored positively and normal mucosa scored negatively (BH-adjusted p = 0.024), whereas iIOs were positioned neutrally (malignancy index ≈ 0) and were found to differ significantly from both the malignant tumor pole (p = 0.027) and the normal-mucosa pole (p = 0.026) (Fig. S3D). The result of this analysis indicates that iIOs do not exhibit a malignant profile and that their proliferative state reflects their crypt/progenitor, fetal-like identity rather than being malignancy-derived. This finding is consistent with their non-malignant, iPSC-derived origin. To further support this interpretation using cohort-external signatures, we scored all samples against a panel of published CRC malignancy proliferation, and oncogenic-driver gene sets (Fig. S5, Table S5). Relative to normal mucosa, iIOs were elevated on proliferation- and crypt/progenitor-associated programs (E2F, G2M, MYC targets, WNT/β-catenin) that are core features of the proliferation crypt stem/progenitor compartment, but not on tumor-specific oncogenic drivers; KRAS-up scores were lower in iIOs than normal mucosa and p53 was unchanged. These results corroborate the malignancy-axis findings independently of the cohort-derived axis. It should be noted that this interpretation is subject to an inherent circularity. The malignancy axis was derived from tumor-versus-normal differences within the same cohort, and projecting iIOs onto this axis does not constitute an independent validation of non-malignant status. Accordingly, the neutral malignancy-axis position of iIOs is interpreted as one of several converging lines of evidence, and definitive assessment of malignant potential relies on the genomic characterization of iPSC lines (normal karyotype, absence of recurrent CRC copy-number alteration, mycoplasma-negative status, integration-free reprogramming).

Finally, our analysis included the examination of the metabolic status between the groups at the transcriptomic level. As a result of these analyses, which were performed based on curated gene lists, iIOs were found to generally display increased metabolic signatures; however, the glycolysis-OXPHOS balance, referred to as the Warburg index, did not differ between groups. When the relationship between composite anabolic metabolism and proliferation was examined in primary tissue biopsies and iIOs, the highest correlation was observed in iIOs (r = 0.89, 95% CI 0.72–0.96, p = 8.9 × 10^−7^, Fig. 7D); at the module level, this coupling was strongest for nucleotide synthesis, the pentose-phosphate pathway, glutaminolysis, and cholesterol biosynthesis, whereas fatty acid oxidation showed a negative correlation (Fig. 7E). In conclusion, the transcriptomic pattern observed in iIOs is consistent with proliferation-associated anabolism supporting biomass accumulation in an expanding culture, rather than autonomous, malignancy-like metabolic reprogramming [49], [50]. We emphasize that this interpretation is based entirely on RNA-level signature scores, orthogonal functional assays including metabolic flux measurements, oxygen consumption rate profiling or stable-isotope tracing will be necessary to confirm reflects actual differences in metabolic activity.

Our multi-layered transcriptomic analyses have underlined that iIOs are valuable epithelial reference systems. iIOs preserve intestinal identity, form organized three-dimensional structures and enable the controlled investigation of epithelial differentiation and developmental patterning. Therefore, they are not models that can replace adult normal mucosa or serve as counterparts to primary CRC biopsies. Since iIOs are not derived from tumor tissue but from iPSCs obtained through the reprogramming of the patient's somatic cells, they cannot be considered a malignant cancer model. The findings obtained within the scope of this study demonstrated for the first time, using samples obtained from the same patients, that iIOs diverge from primary tissue along a developmental rather than an oncogenic axis.

Therefore, iIOs should not be evaluated as models that can replace patients’ healthy tissue biopsies. While primary tissue preserves its multicellular ecosystem, such as immune infiltration and stromal remodeling, organoids are epithelial models that are distant from the tissue context in terms of cellular composition. Particularly in colorectal cancer modeling, patient-derived biopsy-based tumor organoids remain the preferred model because they preserve the histology, genomic features, and drug-response behavior of the tumor from which they originate, thereby providing the system closest to tumor biology [18], [19], [20], [51]. iIOs, on the other hand, should be best positioned as a complementary, developmentally defined normal-epithelial reference. Positioning iIOs within the broader organoid landscape requires distinguishing them from three complementary systems [52]. Patient-derived tumor organoids (PDTOs), established directly from resected tumor tissue, preserve somatic mutation profiles, intratumoral heterogeneity, and drug-response behavior, making them the preferred tool for pharmacological screening and personalized therapy. Adult normal organoids, derived from intestinal crypt stem cells, Recapitulate differentiated epithelial lineages and are suited for barrier function and epithelial maturation studies. Tissue-section models retain the full multicellular microenvironment and are uniquely appropriate for tumor-stroma and immune-evasion studies. iIOs occupy a distinct niche: as isogenic, non-malignant, developmentally defined systems free of pre-existing somatic alterations, their primary value lies in modelling intestinal epithelial development, benchmarking differentiation protocols, and providing a patient-matched non-malignant baseline for controlled perturbation experiments, roles that PDTOs, adult organoids, and tissue sections are not designed to fulfill.

### 4.1 Limitations of the study

Several limitations should be acknowledged. First, the cohort is small (six patients with patient-matched tumor, normal-mucosa, and organoid samples), so the subgroup comparisons and the consensus molecular subtype classification are descriptive rather than definitive [53]. Second, the analyses are based on bulk RNA-seq; although reference-based deconvolution and curated signature scoring supported differences in immune, stromal, and epithelial lineage composition, these approaches are inferential and would benefit from single-cell or spatial validation. Third, the deconvolution reference is epithelial-only; biopsy proportions therefore reflect epithelial transcriptional signal composition rather than absolute cell-type fractions, and the estimates carry inherent uncertainty from reference incompleteness and non-epithelial signal contamination. Fourth, our conclusions are transcriptomic; functional, proteomic, and metabolic assays would be required to confirm the inferred differentiation state, barrier properties, and metabolic dependencies. Fifth, organoids were generated with a single differentiation protocol and analyzed at a single time point (day 120); as discussed above, the degree of fetal-like and progenitor bias may therefore differ in other systems [54], [55]. The malignancy-axis analysis is based on a single cohort-derived axis; although iIOs were neutral on the composite malignancy index and lay orthogonal to the tumor–normal axis, individual oncogenic signature differences were not robust to multiple-testing correction, so the non-malignant interpretation rests on the convergence of these analyses rather than on any single signature. Finally, all metabolic inferences are transcript-based; functional metabolic assays would be required to confirm the directionality and magnitude of the metabolic differences suggested by gene-expression signatures. Somatic variants pre-existing in the parental PBMCs used for reprogramming were not screened by whole-genome or targeted sequencing. The possibility that low-frequency somatic variants were carried over into the iPSC lines cannot be excluded. Additionally, reprogramming-associated epigenetic memory was not formally assessed, although integration-free Sendai virus reprogramming minimizes epigenetic retention of the somatic cell of origin, residual epigenetic memory cannot be ruled out.Considering these limitations, the fact that the findings of seven methodologically independent analyses completed within the scope of the study support each other strengthens the quality of the study.

## Ethics declaration

Written informed consent to take part in the study and to publish the article has been obtained from all participants or their legal representatives. The privacy rights of participants have been observed.

This study included organ or tissue donors. This study includes human biological material and consent was obtained by donors, or their next of kin or legal representatives, for use in this study and for publication of the article. The samples used in this research were not sourced from executed prisoners or prisoners of conscience.

This study was performed in compliance with relevant laws, regulatory frameworks and guidelines where the research took place. This study was approved by the Lokman Hekim University Human Research Ethics Committee. (Approval No. No. 2021/157)

## Author contributions

Zeynep Büşra Aksoy (Z.B.A.): Conceptualization, Methodology, Software, Formal analysis, Investigation, Data curation, Writing – original draft, Writing – review & editing, Visualization. Recep Uyar (R.U.): Methodology, Writing – review & editing. Kamil Can Akçalı; (K.C.A.): Supervision. Hilal Özdağ-Sevgili (H.Ö. S.): Funding acquisition.

## Ethical approval

All procedures involving human samples were conducted in accordance with the Declaration of Helsinki and approved by the Lokman Hekim University Human Research Ethics Committee (Approval Date: 23/12/2021; Decision No: 2021/157). Informed consent was obtained from all participants or their legal guardians.

## Declaration of generative AI and AI-assisted technologies in the manuscript preparation process

During the preparation of this work, the author(s) used Claude (Anthropic) to assist with the implementation of bioinformatic analyses and the generation of figures, specifically the writing and debugging of analysis and plotting code. No generative AI was used to write or edit the text of the manuscript. All AI-assisted code, statistical outputs, and figures were reviewed, verified, and edited by the author(s), who take full responsibility for the content of the published article.

## Materials availability

The human iPSC lines generated in this study are available from the lead contact upon reasonable request.

## Data and code availability statement

The RNA-seq data supporting the conclusions of this article have been deposited in the NCBI Gene Expression Omnibus (GEO) and are accessible through GEO Series accession number GSE347528; bioproject number: PRJNA1465135. All original analysis code has been deposited at Zenodo (DOI: 10.5281/zenodo.21472175) and at GitHub (https://github.com/zeynepbusraaa/iPSC-intestinal-organoid-CRC-transcriptomics), and is publicly available as of the date of publication.

## Declaration of competing interest

The authors declare that they have no known competing financial interests or personal relationships that could have appeared to influence the work reported in this paper.

## Figures and Tables

**figs1:**
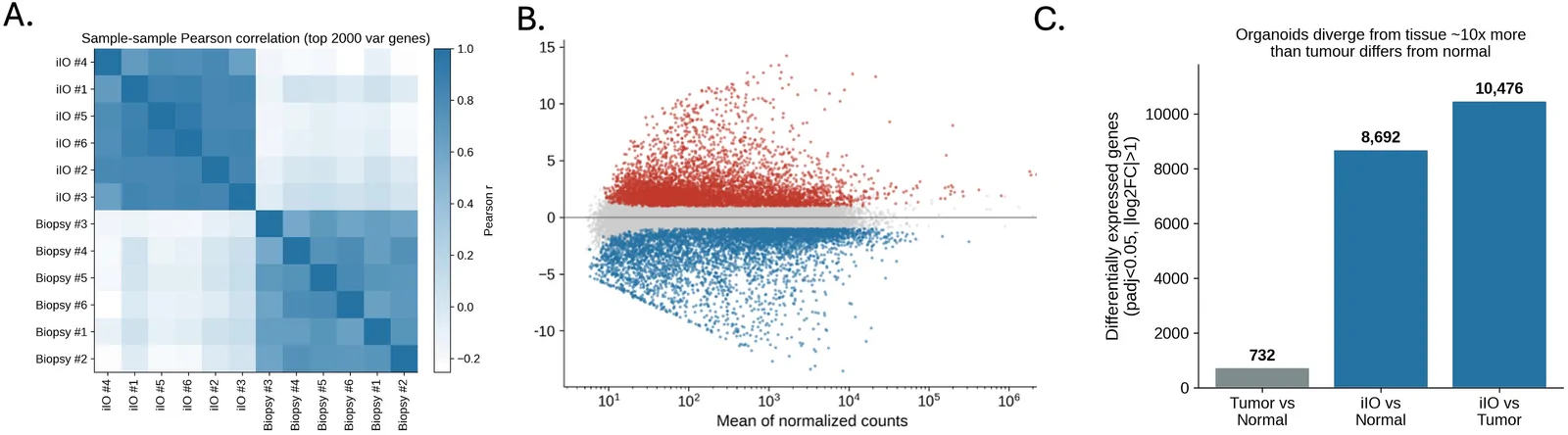


**figs2:**
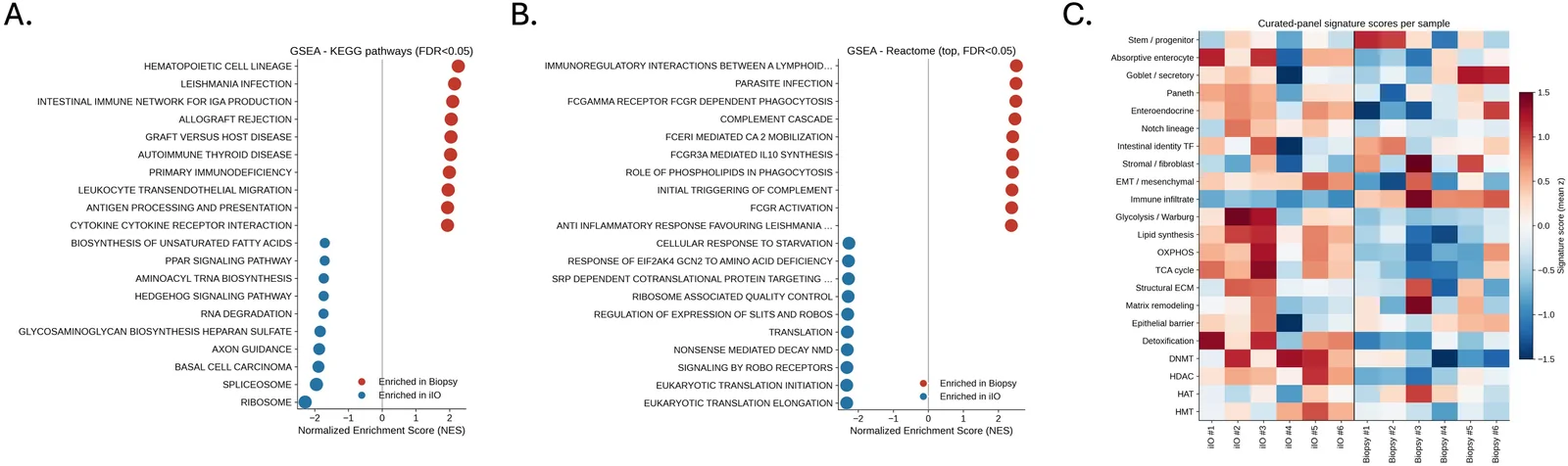


**figs3:**
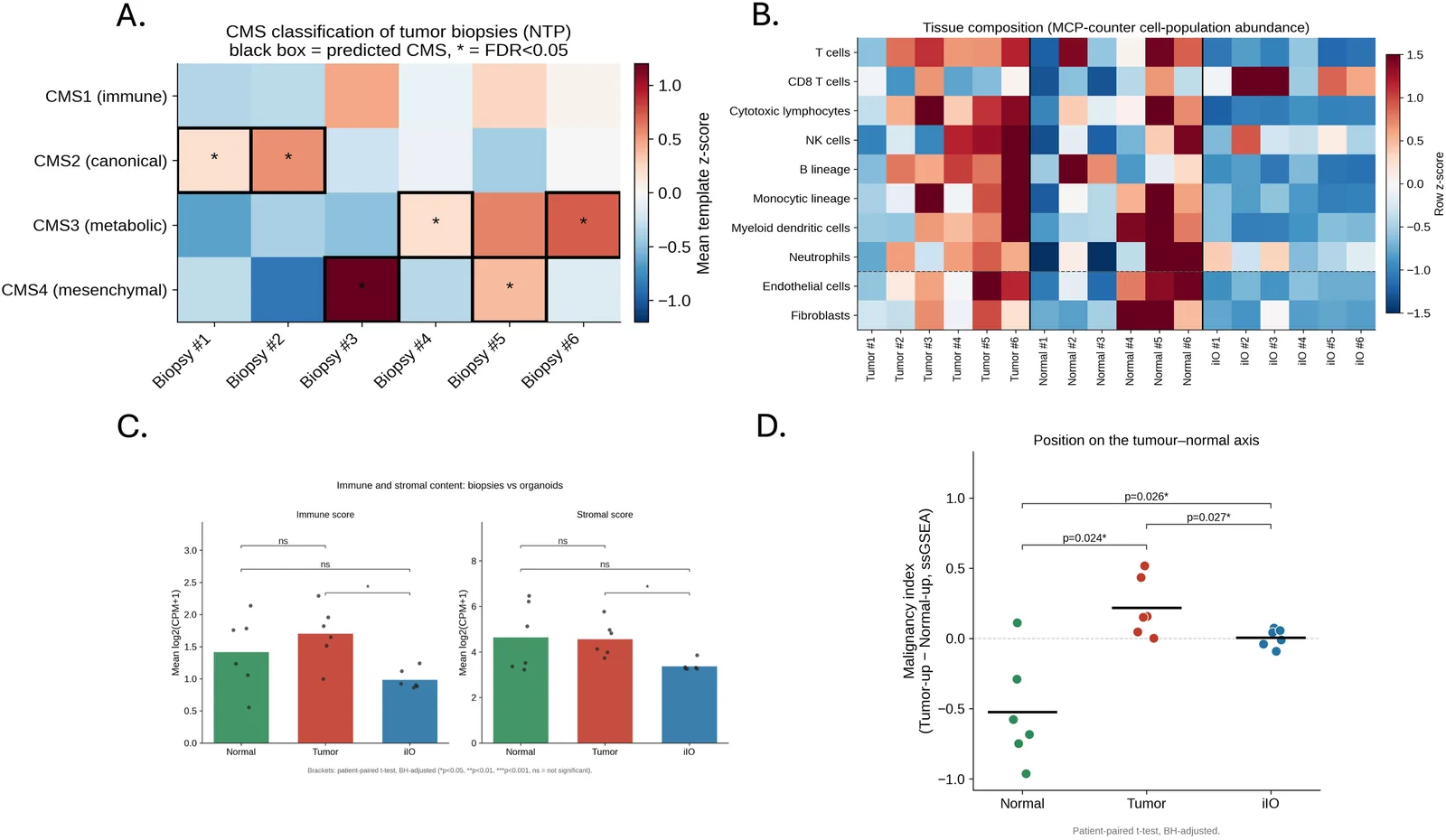


**figs4:**
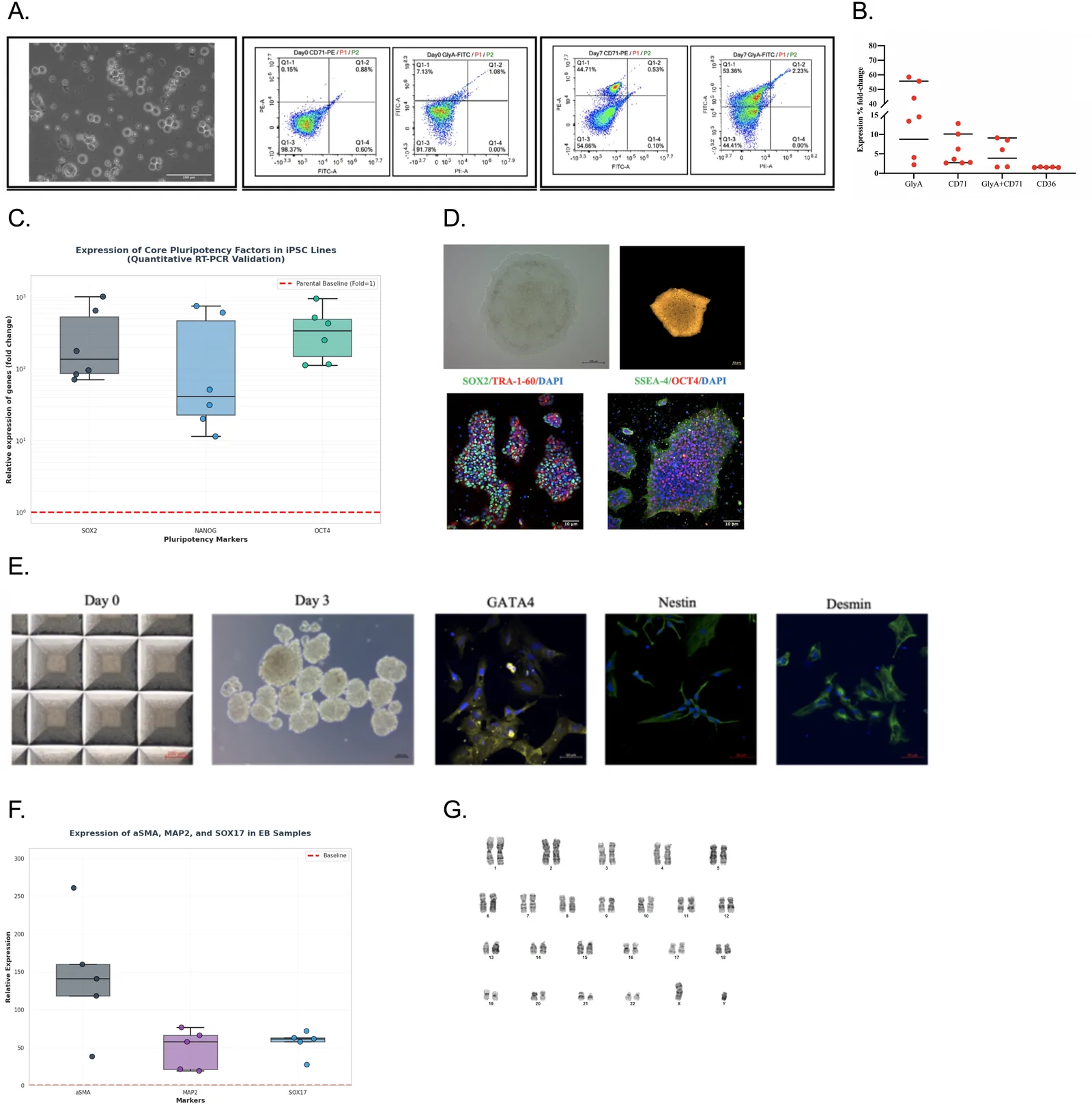


**figs5:**
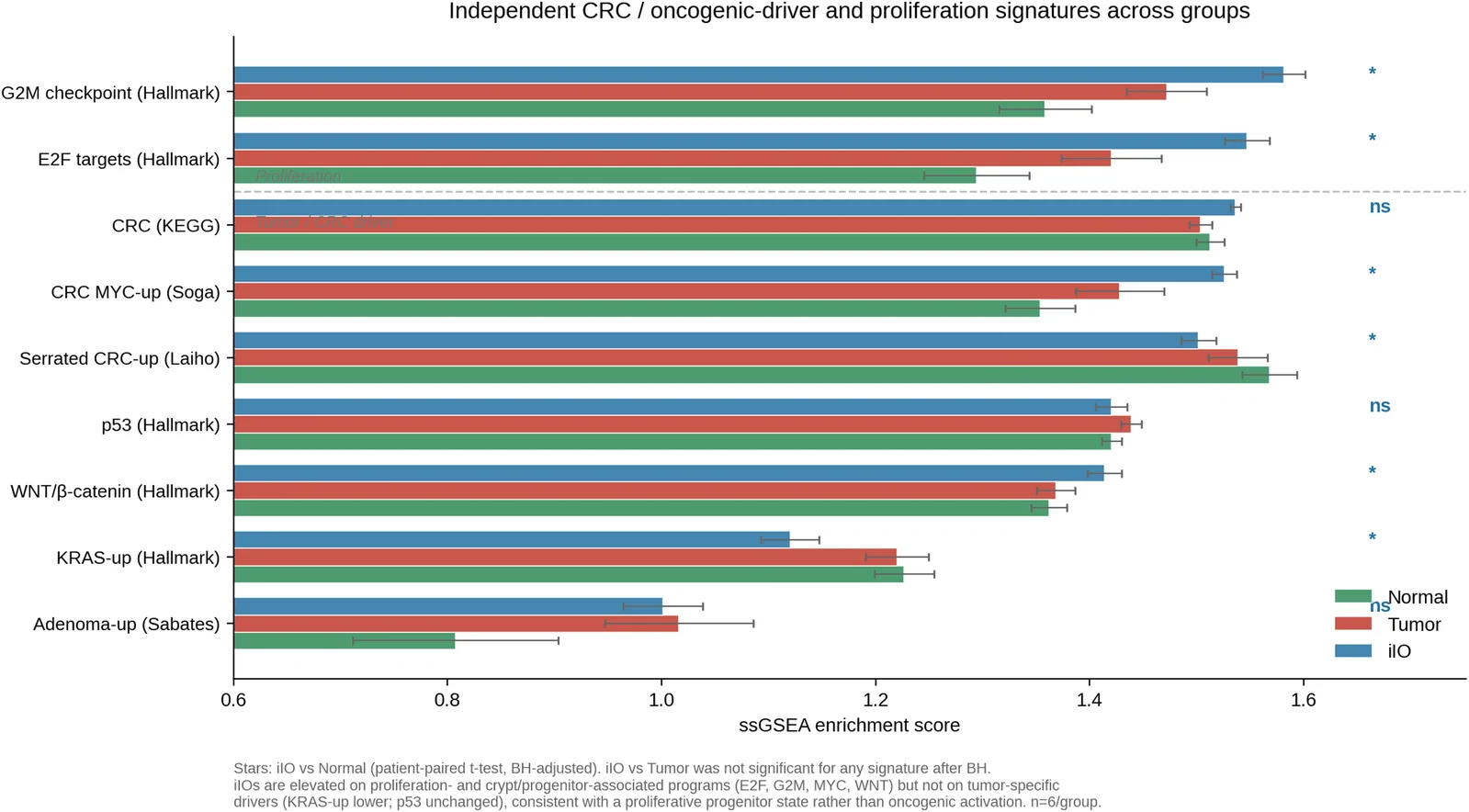


## Data Availability

The RNA-seq data supporting the conclusions of this article have been deposited in the NCBI Gene Expression Omnibus (GEO) GSE347528 and bioproject number: PRJNA1465135.
