# Supplementary material for: Transcriptomic benchmarking positions iPSC-Derived intestinal organoids as immature, non-malignant epithelial references for colorectal cancer tissue
Source: Biochem Biophys Rep. 2026 Nov 3;48:102859. doi: 10.1016/j.bbrep.2026.102859 (PMC13756187; doi:10.1016/j.bbrep.2026.102859)
Supplement: Supplementary file 1 [file mmc2.docx]

**KEY RESOURCES TABLE**

| **REAGENT or RESOURCE** | **SOURCE** | **IDENTIFIER** |
| --- | --- | --- |
| **Antibodies** |  |  |
| Anti-OCT4 (IF) | Invitrogen | Cat# A24867; RRID: N/A |
| Anti-SSEA4 (IF) | Invitrogen | Cat# A24866; RRID: N/A |
| Anti-SOX2 (IF) | Invitrogen | Cat# A24759; RRID: N/A |
| Anti-TRA-1-60 (IF) | Invitrogen | Cat# A24868; RRID: N/A |
| Anti-FOXA2 (IF) | Invitrogen | Cat# 701698; RRID: N/A |
| Anti-KLF5 (IF) | Invitrogen | Cat# PA5-27876; RRID: N/A |
| Anti-Chromogranin A (IF) | Invitrogen | Cat# PA5-35071; RRID: N/A |
| Anti-SOX9 (IF) | Invitrogen | Cat# MA5-41174; RRID: N/A |
| Anti-Lysozyme (IF) | Invitrogen | Cat# MA5-32154; RRID: N/A |
| Anti-Villin (IF) | Invitrogen | Cat# PA5-22072; RRID: N/A |
| Anti-LGR5 (IF) | Elabscience | Cat# E-AB-63432; RRID: N/A |
| Anti-CDX2 (IF) | Elabscience | Cat# E-AB-22020; RRID: N/A |
| Anti-Nestin (IF) | Elabscience | Cat# E-AB-63599; RRID: N/A |
| Anti-Desmin (IF) | Elabscience | Cat# E-AB-31185; RRID: N/A |
| Anti-GATA4 (IF) | Santa Cruz Biotechnology | Cat# sc-25310; RRID: N/A |
| Anti-PDGFRA (flow cytometry) | Cell Signaling Technology | Cat# 8533S; RRID: N/A |
| Anti-CD117 (flow cytometry) | Elabscience | Cat# E-AB-F1150C; RRID: N/A |
| Anti-CXCR4 (flow cytometry) | Elabscience | Cat# E-AB-F1157D; RRID: N/A |
| FITC isotype control (flow cytometry) | BD Biosciences | Cat# 555583; RRID: N/A |
| PE isotype control (flow cytometry) | BD Biosciences | Cat# 555574; RRID: N/A |
| **Biological samples** |  |  |
| Primary CRC tumor biopsies (n = 6) | This study; Lokman Hekim University | Ethics No. 2021/157 |
| Patient-matched normal colonic mucosa (n = 6) | This study; Lokman Hekim University | Ethics No. 2021/157 |
| **Critical commercial assays** |  |  |
| Watchmaker RNA Library Prep Kit with Polaris Depletion | Watchmaker Genomics | Cat# 7BK0002 |
| **Deposited data** |  |  |
| RNA-seq data (this study) | This study | GEO: GSEXXXXXX; BioProject: PRJNA1465135 |
| Human Gut Cell Atlas | Elmentaite et al.¹ | <https://www.gutcellatlas.org> |
| MSigDB (Hallmark, KEGG, Reactome) | Liberzon et al.² | RRID:SCR_016863 |
| **Experimental models: Cell lines** |  |  |
| Human iPSC lines (reprogrammed from erythroid progenitor cells by using Sendai virüs), n = 6 | This study | iPSC-CRC1, iPSC-CRC2, iPSC-CRC3, iPSC-mCRC1, iPSC-mCRC2, iPSC-mCRC3 |
| **Oligonucleotides** |  |  |
| RT-qPCR primers (see Methods, RT-PCR section): β-Actin, OCT4, SOX2, NANOG, c-MYC, KLF4, αSMA, SOX17, MAP2 | This study | Methods, RT-PCR section |
| **Software and algorithms** |  |  |
| FastQC | Andrews | RRID:SCR_014583 |
| Cutadapt | Martin | RRID:SCR_011841 |
| STAR (2-pass) | Dobin et al. | RRID:SCR_004463 |
| DESeq2 | Love et al. | RRID:SCR_015687 |
| PyDESeq2 | Muzellec et al. | <https://github.com/owkin/PyDESeq2> |
| GSEA / gseapy | Subramanian et al. | RRID:SCR_003199 |
| CMScaller | Eide et al. | <https://github.com/Lothelab/CMScaller> |
| MCP-counter | Becht et al. | <https://github.com/ebecht/MCPcounter> |
| Python 3.12 | Python Software Foundation | RRID:SCR_008394 |
| R | R Core Team | RRID:SCR_001905 |
| Original analysis code (this study) | This study | [DOI: 10.5281/zenodo.21472175](https://doi.org/10.5281/zenodo.21472175) |
