# Supplementary material for: Transcriptomic benchmarking positions iPSC-Derived intestinal organoids as immature, non-malignant epithelial references for colorectal cancer tissue
Source: Biochem Biophys Rep. 2026 Nov 3;48:102859. doi: 10.1016/j.bbrep.2026.102859 (PMC13756187; doi:10.1016/j.bbrep.2026.102859)
Supplement: Supplementary file 2 [file mmc6.docx]

# **Supplementary QC File, Figure Legend**

# **All quality control figures follow the same panel layout for each iPSC line:**

# **(A)** Immunofluorescence confirmation of pluripotency markers. SSEA-4 (Alexa Fluor 488, green) and OCT4 (Alexa Fluor 555, red) co-expression is shown in the left panel; SOX2 (Alexa Fluor 488, green) and TRA-1-60 (Alexa Fluor 555, red) co-expression in the right panel. Nuclei counterstained with DAPI (blue).

# **(B)** Trilineage differentiation capacity confirmed by immunofluorescence. GATA4 (AF 488, yellow) marks endoderm; Nestin (AF 488, green) marks ectoderm; Desmin (AF 488, green) marks mesoderm.

# **(C)** Sendai vector clearance confirmed by RT-PCR at passage 12 using CytoTune 2.0 primer sets targeting KOS, KLF4, SeV genome, and c-MYC. Positive control (left), iPSC line (centre), and no-template control (right) are shown. Absence of specific amplification in the iPSC panel confirms vector clearance at the time of testing; all organoid differentiations were initiated at passage 20 or later.

# **(D)** Mycoplasma testing by PCR-based detection assay. All six iPSC lines (#1–#6), positive control, and negative control are shown on a single gel. No mycoplasma-specific band is detected in any iPSC line.

# **(E)** G-banding karyotype analysis confirming normal chromosomal complement (46,XY or 46,XX) with no gross structural abnormalities or recurrent CRC-associated copy-number alterations.

# **iPSC Line Quality Control — Patient #1**

| **Patient & Clinical Summary** | |
| --- | --- |
| **Patient No.** | #1 |
| **Sex** | Male |
| **Age at diagnosis** | 70 years |
| **Diagnosis** | Colon adenocarcinoma |
| **Pathological stage** | pT3N0 |
| **MSI status** | MSS |
| **Histological grade** | Grade 2 |

| **iPSC Line Quality Control Parameters** | |
| --- | --- |
| **Clone selection** | Single iPSC clone selected (passage 5) per patient based on morphology, growth rate, and pluripotency marker expression (SSEA-4, OCT4, Tra-1-60, SOX2, NANOG (qPCR)) |
| **Reprogramming method** | Integration-free Sendai virus (CytoTune-iPS 2.0); peripheral blood mononuclear cells (PBMCs) used as starting material |
| **Sendai virus clearance** | Confirmed absent by RT-PCR at passage ≥12; no residual vector signal detected |
| **Karyotype / CNV status** | Normal karyotype (46,XY or 46,XX); no recurrent CRC-associated copy-number alterations detected by G-banding analysis |
| **CRC driver gene status** | No known CRC driver mutations (APC, KRAS, TP53, SMAD4) introduced by reprogramming; somatic variants of the parental PBMC not screened by WGS (acknowledged as limitation) |
| **Mycoplasma testing** | Negative; tested by PCR-based mycoplasma detection assay prior to differentiation |
| **Pluripotency markers** | OCT4, NANOG, SOX2 confirmed by immunofluorescence; SSEA-4 confirmed by flow cytometry |
| **Trilineage differentiation** | Confirmed by directed differentiation into ectoderm, mesoderm, and endoderm lineages; representative images provided in Fig. S4 |
| **Epigenetic memory** | Not formally assessed; acknowledged as limitation in the manuscript. Integration-free reprogramming minimizes, but does not eliminate, epigenetic memory of the somatic cell of origin |
| **Healthy-donor control** | Not included in the current study; acknowledged as limitation. Patient-matched iPSCs serve as the isogenic non-malignant reference baseline |

# **iPSC Line Quality Control — Patient #2**

| **Patient & Clinical Summary** | |
| --- | --- |
| **Patient No.** | #2 |
| **Sex** | Male |
| **Age at diagnosis** | 58 years |
| **Diagnosis** | Colon adenocarcinoma |
| **Pathological stage** | pT3N1 |
| **MSI status** | MSS |
| **Histological grade** | Grade 1 |

| **iPSC Line Quality Control Parameters** | |
| --- | --- |
| **Clone selection** | Single iPSC clone selected (passage 5) per patient based on morphology, growth rate, and pluripotency marker expression (SSEA-4, OCT4, Tra-1-60, SOX2, NANOG (qPCR)) |
| **Reprogramming method** | Integration-free Sendai virus (CytoTune-iPS 2.0); peripheral blood mononuclear cells (PBMCs) used as starting material |
| **Sendai virus clearance** | Confirmed absent by RT-PCR at passage ≥12; no residual vector signal detected |
| **Karyotype / CNV status** | Normal karyotype (46,XY or 46,XX); no recurrent CRC-associated copy-number alterations detected by G-banding analysis |
| **CRC driver gene status** | No known CRC driver mutations (APC, KRAS, TP53, SMAD4) introduced by reprogramming; somatic variants of the parental PBMC not screened by WGS (acknowledged as limitation) |
| **Mycoplasma testing** | Negative; tested by PCR-based mycoplasma detection assay prior to differentiation |
| **Pluripotency markers** | OCT4, NANOG, SOX2 confirmed by immunofluorescence; SSEA-4 confirmed by flow cytometry |
| **Trilineage differentiation** | Confirmed by directed differentiation into ectoderm, mesoderm, and endoderm lineages; representative images provided in Fig. S4 |
| **Epigenetic memory** | Not formally assessed; acknowledged as limitation in the manuscript. Integration-free reprogramming minimizes, but does not eliminate, epigenetic memory of the somatic cell of origin |
| **Healthy-donor control** | Not included in the current study; acknowledged as limitation. Patient-matched iPSCs serve as the isogenic non-malignant reference baseline |

# **iPSC Line Quality Control — Patient #3**

| **Patient & Clinical Summary** | |
| --- | --- |
| **Patient No.** | #3 |
| **Sex** | Female |
| **Age at diagnosis** | 63 years |
| **Diagnosis** | Colon adenocarcinoma |
| **Pathological stage** | pT4aN1a |
| **MSI status** | MSS |
| **Histological grade** | Grade 3 |

| **iPSC Line Quality Control Parameters** | |
| --- | --- |
| **Clone selection** | Single iPSC clone selected (passage 5) per patient based on morphology, growth rate, and pluripotency marker expression (SSEA-4, OCT4, Tra-1-60, SOX2, NANOG (qPCR)) |
| **Reprogramming method** | Integration-free Sendai virus (CytoTune-iPS 2.0); peripheral blood mononuclear cells (PBMCs) used as starting material |
| **Sendai virus clearance** | Confirmed absent by RT-PCR at passage ≥12; no residual vector signal detected |
| **Karyotype / CNV status** | Normal karyotype (46,XY or 46,XX); no recurrent CRC-associated copy-number alterations detected by G-banding analysis |
| **CRC driver gene status** | No known CRC driver mutations (APC, KRAS, TP53, SMAD4) introduced by reprogramming; somatic variants of the parental PBMC not screened by WGS (acknowledged as limitation) |
| **Mycoplasma testing** | Negative; tested by PCR-based mycoplasma detection assay prior to differentiation |
| **Pluripotency markers** | OCT4, NANOG, SOX2 confirmed by immunofluorescence; SSEA-4 confirmed by flow cytometry |
| **Trilineage differentiation** | Confirmed by directed differentiation into ectoderm, mesoderm, and endoderm lineages; representative images provided in Fig. S4 |
| **Epigenetic memory** | Not formally assessed; acknowledged as limitation in the manuscript. Integration-free reprogramming minimizes, but does not eliminate, epigenetic memory of the somatic cell of origin |
| **Healthy-donor control** | Not included in the current study; acknowledged as limitation. Patient-matched iPSCs serve as the isogenic non-malignant reference baseline |

# **iPSC Line Quality Control — Patient #4**

| **Patient & Clinical Summary** | |
| --- | --- |
| **Patient No.** | #4 |
| **Sex** | Male |
| **Age at diagnosis** | 64 years |
| **Diagnosis** | Colon adenocarcinoma |
| **Pathological stage** | pT3N0M1 |
| **MSI status** | MSS |
| **Histological grade** | Grade 1 |

| **iPSC Line Quality Control Parameters** | |
| --- | --- |
| **Clone selection** | Single iPSC clone selected (passage 5) per patient based on morphology, growth rate, and pluripotency marker expression (SSEA-4, OCT4, Tra-1-60, SOX2, NANOG (qPCR)) |
| **Reprogramming method** | Integration-free Sendai virus (CytoTune-iPS 2.0); peripheral blood mononuclear cells (PBMCs) used as starting material |
| **Sendai virus clearance** | Confirmed absent by RT-PCR at passage ≥12; no residual vector signal detected |
| **Karyotype / CNV status** | Normal karyotype (46,XY or 46,XX); no recurrent CRC-associated copy-number alterations detected by G-banding analysis |
| **CRC driver gene status** | No known CRC driver mutations (APC, KRAS, TP53, SMAD4) introduced by reprogramming; somatic variants of the parental PBMC not screened by WGS (acknowledged as limitation) |
| **Mycoplasma testing** | Negative; tested by PCR-based mycoplasma detection assay prior to differentiation |
| **Pluripotency markers** | OCT4, NANOG, SOX2 confirmed by immunofluorescence; SSEA-4 confirmed by flow cytometry |
| **Trilineage differentiation** | Confirmed by directed differentiation into ectoderm, mesoderm, and endoderm lineages; representative images provided in Fig. S4 |
| **Epigenetic memory** | Not formally assessed; acknowledged as limitation in the manuscript. Integration-free reprogramming minimizes, but does not eliminate, epigenetic memory of the somatic cell of origin |
| **Healthy-donor control** | Not included in the current study; acknowledged as limitation. Patient-matched iPSCs serve as the isogenic non-malignant reference baseline |

# **iPSC Line Quality Control — Patient #5**

| **Patient & Clinical Summary** | |
| --- | --- |
| **Patient No.** | #5 |
| **Sex** | Female |
| **Age at diagnosis** | 56 years |
| **Diagnosis** | Colorectal adenocarcinoma |
| **Pathological stage** | pT3N1 |
| **MSI status** | MSS |
| **Histological grade** | Grade 1 |

| **iPSC Line Quality Control Parameters** | |
| --- | --- |
| **Clone selection** | Single iPSC clone selected (passage 5) per patient based on morphology, growth rate, and pluripotency marker expression (SSEA-4, OCT4, Tra-1-60, SOX2, NANOG (qPCR)) |
| **Reprogramming method** | Integration-free Sendai virus (CytoTune-iPS 2.0); peripheral blood mononuclear cells (PBMCs) used as starting material |
| **Sendai virus clearance** | Confirmed absent by RT-PCR at passage ≥12; no residual vector signal detected |
| **Karyotype / CNV status** | Normal karyotype (46,XY or 46,XX); no recurrent CRC-associated copy-number alterations detected by G-banding analysis |
| **CRC driver gene status** | No known CRC driver mutations (APC, KRAS, TP53, SMAD4) introduced by reprogramming; somatic variants of the parental PBMC not screened by WGS (acknowledged as limitation) |
| **Mycoplasma testing** | Negative; tested by PCR-based mycoplasma detection assay prior to differentiation |
| **Pluripotency markers** | OCT4, NANOG, SOX2 confirmed by immunofluorescence; SSEA-4 confirmed by flow cytometry |
| **Trilineage differentiation** | Confirmed by directed differentiation into ectoderm, mesoderm, and endoderm lineages; representative images provided in Fig. S4 |
| **Epigenetic memory** | Not formally assessed; acknowledged as limitation in the manuscript. Integration-free reprogramming minimizes, but does not eliminate, epigenetic memory of the somatic cell of origin |
| **Healthy-donor control** | Not included in the current study; acknowledged as limitation. Patient-matched iPSCs serve as the isogenic non-malignant reference baseline |

# **iPSC Line Quality Control — Patient #6**

| **Patient & Clinical Summary** | |
| --- | --- |
| **Patient No.** | #6 |
| **Sex** | Male |
| **Age at diagnosis** | 73 years |
| **Diagnosis** | Colorectal adenocarcinoma |
| **Pathological stage** | pT3N1c |
| **MSI status** | MSS |
| **Histological grade** | Grade 2 |

| **iPSC Line Quality Control Parameters** | |
| --- | --- |
| **Clone selection** | Single iPSC clone selected (passage 5) per patient based on morphology, growth rate, and pluripotency marker expression (SSEA-4, OCT4, Tra-1-60, SOX2, NANOG (qPCR)) |
| **Reprogramming method** | Integration-free Sendai virus (CytoTune-iPS 2.0); peripheral blood mononuclear cells (PBMCs) used as starting material |
| **Sendai virus clearance** | Confirmed absent by RT-PCR at passage ≥12; no residual vector signal detected |
| **Karyotype / CNV status** | Normal karyotype (46,XY or 46,XX); no recurrent CRC-associated copy-number alterations detected by G-banding analysis |
| **CRC driver gene status** | No known CRC driver mutations (APC, KRAS, TP53, SMAD4) introduced by reprogramming; somatic variants of the parental PBMC not screened by WGS (acknowledged as limitation) |
| **Mycoplasma testing** | Negative; tested by PCR-based mycoplasma detection assay prior to differentiation |
| **Pluripotency markers** | OCT4, NANOG, SOX2 confirmed by immunofluorescence; SSEA-4 confirmed by flow cytometry |
| **Trilineage differentiation** | Confirmed by directed differentiation into ectoderm, mesoderm, and endoderm lineages; representative images provided in Fig. S4 |
| **Epigenetic memory** | Not formally assessed; acknowledged as limitation in the manuscript. Integration-free reprogramming minimizes, but does not eliminate, epigenetic memory of the somatic cell of origin |
| **Healthy-donor control** | Not included in the current study; acknowledged as limitation. Patient-matched iPSCs serve as the isogenic non-malignant reference baseline |
